# Microgrids as a Tool for Energy Self-Sufficiency

**DOI:** 10.3390/s25216707

**Published:** 2025-11-02

**Authors:** Sławomir Bielecki, Tadeusz Skoczkowski, Marcin Wołowicz

**Affiliations:** Faculty of Power and Aeronautical Engineering, Warsaw University of Technology, 21/25 Nowowiejska St., 00-665 Warsaw, Poland; tadeusz.skoczkowski@pw.edu.pl (T.S.); marcin.wolowicz@pw.edu.pl (M.W.)

**Keywords:** microgrids, smart grid, decarbonisation, energy management system, energy storage, distributed generation, ancillary services

## Abstract

The article presents an overview of knowledge in the field of energy microgrids as smart structures enabling energy self-sufficiency, with particular emphasis on decarbonisation. Based on a review of the literature and technical solutions, the characteristics have been classified and, emphasising the potential for integrating different technologies within microgrid structures, the role that microgrids and their users can play in the functioning of the energy system has been defined. Energy microgrids can be the pillar on which smart energy structures and smart grids, including energy systems using multiple energy carriers, will be based. Microgrids can guarantee energy self-sufficiency within their area of operation and support the entire energy system in this respect. Sensors that respond to both electrical and non-electrical quantities must play a special role in such structures, as they form the technical basis for the functioning of the smart energy sector.

## 1. Introduction

Microgrids are currently regarded as an element of modern, transforming energy systems. They are associated with concepts such as microgeneration, distributed generation, renewable energy sources, energy storage, energy management, demand response, and above all, smart grids.

The history of energy microgrids dates back to the beginning of the history of electrical power engineering. The first structure supplying electricity using DC systems, built by T. Edison in 1882, was in fact a microgrid [1,2].

Small systems were also the starting point for the energy industry in other parts of the world. In Europe, in 1882, De Bolltes supplied 1.5 kW generated by a steam engine at the Miesbach coal mine to an area 57 km away via a direct current line at a voltage that today would be classified as medium voltage [3]. In 1897, the Sidrapong hydroelectric power station in India was commissioned to serve local loads [4]. Small systems were gradually connected into a larger network, creating more complex power systems operating synchronously and now covering entire continents. The modern network operating within the power system is one of the greatest achievements of modern engineering, a huge, man-made machine on an intercontinental scale with thousands of generators and millions of customers [5].

After the famous blackout in the USA in 2003 and later after Hurricane Sandy in 2012, installations with diesel generators as a backup power source began to gain popularity, enabling effective power supply to facilities for a longer period of time without the need to draw energy from the grid [1,6].

**Figure 1:**
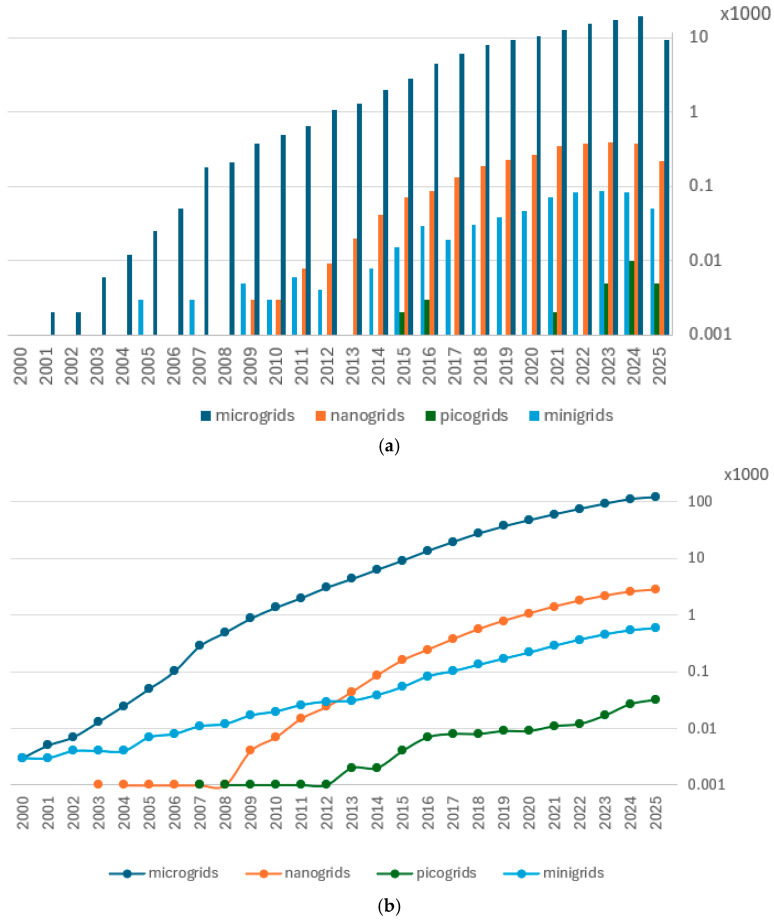
Number of articles (in thousands, logarithmic scale) in the Scopus database in the field of energy (SUBJAREA(ENER)) concerning ‘microgrids’ (or ‘microgrid’); ‘nanogrids’ (or ‘nanogrid’); picogrids (or ‘picogrid’), published in subsequent years—annual increases (**a**), cumulative values (**b**) as of 27 July 2025.

Such structures can be considered microgrids. History thus comes full circle. Interest in the concept of microgrids and its clear development came after the first decade of the 21st century. This development was driven by advances in renewable energy technologies, small-scale generation, energy storage systems and power electronics [7,8]. The result of the growing popularity and development of the microgrid concept during this period is a non-linear increase in the number of scientific publications in the field of energy containing the keyword ‘microgrid’ (Figure 1).

The scale of scientific interest in the area of distributed energy systems is clearly focused on microgrids, which are seen as the most versatile and scalable solution. The number of publications using the concept of microgrids is by far the largest, with more than 123,000 papers in 2025. Since 2008, we have seen an exponential increase in interest in this issue. Nanogrids and minigrids are gaining attention as solutions for specific contexts, and although the number of publications using these concepts is steadily increasing (reaching around 3000 and 60 entries, respectively, in 2025), it is orders of magnitude smaller compared to publications on microgrids. Picogrids remain in the realm of conceptual considerations and do not have a significant impact on mainstream energy research (approximately 30 publications by 2025)—Figure 1.

**Figure 2:**
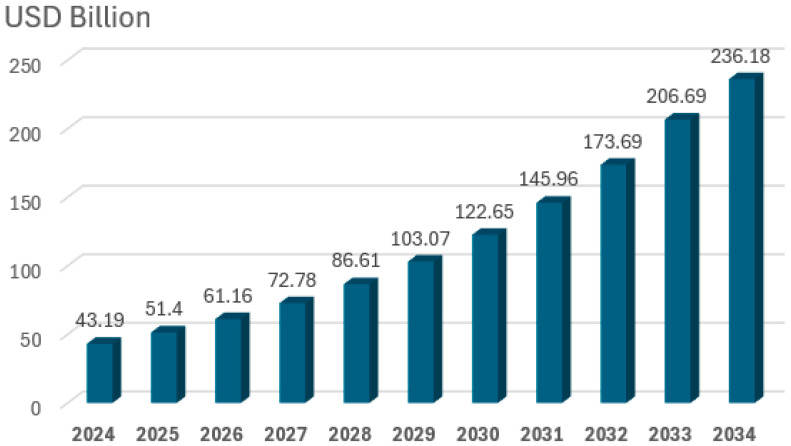
Number Microgrid global market size to 2034 (according to [12]).

Due to the individual and distributed character of projects, the small size of a single investment, the lack of mandatory reporting and, in addition, the lack of a universal definition and thus the lack of a uniform methodology, detailed and up-to-date global statistics on microgrid deployments are not widely available. In 2019, they were estimated to number more than 4500 [9]. There are also discrepancies in estimates of the size of the global microgrid market. According to [10] the global microgrid market was valued at USD 22.9 billion in 2024. The market is expected to grow from USD 28.9 billion in 2025 to USD 140.7 billion in 2034, at a CAGR of 19.2%. According to [11], the global Microgrid market size is USD 42.6 billion in 2024 and will expand at a compound annual growth rate (CAGR) of 21.6% from 2024 to 2031. According to [12] experts predict the global microgrid market will expand significantly, from $43.19 billion in 2024 (share of North America: 41%, Europe: 28%, Azja Pacific: 23%) to approximately $236.18 billion by 2034 (Figure 2). This growth represents a CAGR of 18.52% over the decade, which demonstrates the growing importance of microgrids in the global energy sector. Such developments are driven by, among other things, the growing demand for renewable energy sources, the need to increase the resilience of energy infrastructure and the development of energy storage technologies and grid management systems.

It is worth noting that the rate of growth accelerates in the second half of the decade under review, with the market growing by more than USD 30 billion per year after 2030. This may indicate accelerated global microgrid adoption and increased investment in the energy sector (Figure 2).

Microgrids are becoming a key element in the global energy transition, with their number and installed capacity growing at an impressive rate. The increase in the number and installed capacity of microgrids reflects the growing demand for local, flexible energy solutions.

The aim of this article is to present and analyse knowledge about specific structures, namely energy microgrids, in the context of the energy sector’s transition towards solutions based on zero-emission generation, with particular emphasis on the role of various sensors in the implementation of smart grid solutions. The review methodology consisted of searching scientific databases and conference materials. From these searches, the most relevant and recent items on the functioning and management of microgrids were selected. The search focused on the basic components of microgrids, with particular emphasis on new trends in energy management, enabling energy self-sufficiency, taking into account the issue of decarbonisation.

A narrative literature review methodology was chosen. This approach seems to us to be more appropriate for this topic, given the multifaceted nature that has grown up around the issue of the development of microgrid structures. The criteria for inclusion of publications in this review consisted of two conjunctive conditions, namely: (1) must refer to the concept of microgrids; (2) must come from reliable sources (scientific literature and grey literature, but in the form of industry organisation reports, articles from recognised industry portals, government documents and standards); (3) must address the issue of finding an efficient form of energy distribution and use with a view to ensuring energy sufficiency. In terms of condition (3), more detailed criteria were formulated for the content on an alternative operator basis, namely concerning: (a) the history of the development of energy structures; (b) the definition of concepts; (c) systematics and structure types; (d) structure construction and components; (e) energy management; (f) sensors; (g) decarbonisation and energy transition; (h) energy technology integration; (i) energy self-sufficiency. A departure from direct reference to the concept of microgrids in the cited publications occurred in the part of the article discussing examples of sensor applications to illustrate existing solutions that could potentially also be used in microgrid structures.

Using a Boolean search strategy, the search terms used to select items are (microgrid*) AND (power OR energy) AND (histor* OR definition* OR systematic* OR topolog* OR example* OR component* OR structure* OR formation* OR management* OR sensor* OR decarboniation* OR “energy transformation” OR integration OR self-sufficeincy). A literature search was conducted in the Scopus database. The selection of publications was limited to items no older than 2004 due to the significant increase in popularity of the idea of microgrids that has occurred in the last 20 years.

The selection process involved restricting the items to English language, energy area and open access, yielding almost 33,000 items. From this database of items, using the tool available in Scopus “Refine search”, further keywords related to the themes discussed in the paper were entered, yielding a few to several dozen publications each time, from which titles and abstracts were searched, followed by full texts. In situations where publications referred to other works or documents, the content was confronted with grey literature or other works. Based on the collected materials, an analysis of the situation and the expected role of microgrid-based structures was carried out.

Of the many issues covered, microgrid review papers tend to focus mainly on issues of schemes, mechanisms and control strategies for microgrids ([8,13,14,15,16]), energy management in microgrids ([17,18,19,20]) microgrid architectures ([17,21,22]), economic and market considerations ([13,19,21]) the specifics and need for microgrid interoperability with energy storage systems ([15,16,18,23]) RES generation technology issues in microgrids ([14,15,17,19,20]) equipment, security and/or communication methods in microgrids ([15,16,18,21,22]). The works cited do not address the use of sensors in microgrids more extensively. An example of the few review articles comprehensively addressing the use of sensors in the energy management process in microgrids is the paper [24], which does not address the role and importance of microgrids, nor does its scope include a discussion of microgrid functionality. No publications have been encountered in the literature that critically address the definition and classification of structures commonly referred to as microgrids. This article, in contrast to the aforementioned ones, seeks to present and complete the current picture of the key components of the microgrid category as a modern, intelligent and self-sufficient energy structure, a structure adapted to the challenges of the energy transition. The comprehensiveness of this review relates to the level of potential frameworks for energy self-sufficiency of microgrids based on the use of a variety of sensors.

The article is structured as follows: after the introduction (Section 1), the problem of defining the concept of microgrids is analysed (Section 2), followed by a discussion of possible classifications of microgrids (Section 3). Section 4 analyses the basic components that make up the structure of a microgrid. Section 5 is dedicated to the issue of using sensors in microgrids. Section 6 analyses the functioning of microgrids in the context of the challenges associated with the energy transition, with particular emphasis on decarbonisation. Section 7, based on the information presented in the previous sections, discusses the expected role that microgrids and their users may play in a smart power system with signalling of research directions. The whole is concluded by Section 8 with summary conclusions.

## 2. Definition of a Microgrid

In addition to microgrids, structures comprising smaller installations and energy networks are also referred to as nanogrids, picogrids [25] and minigrids. Picogrids, nanogrids, and microgrids are types of electrical grids typically associated with individual households, buildings, and neighbourhoods, respectively, and are ultimately connected to the main power distribution grid or to another microgrid [21].

A picogrid is a small-scale energy network that distributes power at the device level, utilising devices with storage capabilities as decentralised energy storage units. One key advantage of picogrids is their ability to supply power specifically suited to the requirements of individual appliances [26,27].

Nanogrid refers to a single small power system with features such as: residential, small industrial or commercial site; small in size (<50 kW); behind a single meter; includes the generating source, in-house distribution, and energy storage functions (thermal/electric), also obtained with electric vehicles [28].

The energy distribution system for a single house/building can be called a ‘nanogrid’, while the distribution system for multiple houses/buildings can be called a ‘microgrid’, consisting of interconnected nanogrids [29]. A nanogrid is a compact version of a microgrid, usually designed to supply power to a single building or individual load. Whereas microgrids function as foundational components of a smart grid, nanogrids act as the cell units within a microgrid [30]. Minigrids are small-scale power generation systems that supply electricity to a limited group of users. Their generation capacity typically ranges between 50 kW and 1 MW. They offer an effective solution for providing electricity in rural areas [30].

However, there is currently no consensus on the precise terminology and categorisation of concepts, especially in terms of the installed capacity of structures assigned to pico-, nano-, micro- and milli-grids [21]. The most popular concept in this context is microgrid, and attempts are being made to fully define and standardise it. Microgrids are the subject of dedicated standards (e.g., IEC TS 62898 series). Although there is still no consensus on a strict definition of microgrids in terms of control, operation, and energy/power management [31].

The IEC defines a microgrid as ‘‘group of interconnected loads and distributed energy resources (DER) with clearly defined electrical boundaries that acts as a single controllable entity with respect to the grid and can connect and disconnect from the grid to enable it to operate in both grid-connected or island mode.” [32].

CIGRÉ C6.22 Working Group’s Microgrid Evolution Roadmap, IEEE standard 2030.7 [33] and U.S. Department of Energy (DOE) all define microgrid in similar terms—loads, distributed energy resources (generation, storage and load control), and the concept of operating with or without a grid [34,35,36,37].

The IEEE 2030.7-2017 standard reduces the complexity of microgrids to two fixed operating modes (Steady State Grid Connected and Stable Island) and four types of transitions (Transition from Grid Connected to Steady State Island—Planned; Grid Connected to Steady State Island—Unplanned; Steady State Island reconnect to Grid; Black Start into Steady State Island). These definitions do not mention a control unit for facilitating the system’s features (e.g., seamless transfer and working as a supplementary power supply) [38]. None of the sector definitions specifies the minimum time for which a microgrid should be able to operate efficiently without being connected to the power grid. Nor do the definitions say anything about the size of the distributed energy resources used or the types of technology that may be implemented.

It can be concluded that microgrids are distributed energy resources that can operate autonomously. Microgrids are defined by their function, not by their size. The size of a microgrid depends basically on the peak power required by the loads [39]. Microgrids are anticipated to include all the fundamental elements of a conventional power system on a smaller scale, excluding certain non-essential components like transmission lines and substations. The primary concept behind a microgrid is to integrate a limited number of distributed generation units and manage them efficiently without forming a complicated network. Key elements of a microgrid include a hierarchical control structure, a point of common coupling (PCC), distributed control based on local data, and a defined area that facilitates the organised integration of distributed generation units to ensure the system operates reliably [38].

A microgrid is a possible future energy system paradigm formed by the interconnection of small, modular generation units, storage devices and controllable loads in distribution systems [40]. Microgrids offer an effective platform for energy harvesting and load-side energy management, contributing to energy savings [41].

In view of the above definitions, a microgrid should be treated as a structure enabling the distribution of energy within a specific limited area, which may include various carriers and forms of useful energy, at least partly originating from its own generation sources classified as distributed generation or microgeneration technologies. A microgrid can be equated with a small, physically connected, fully functional, self-controlling and autonomously operating energy system, which may or may not have an active connection to the external national power system. The term ‘small’ system is vague, and in the absence of criteria currently agreed upon by energy experts (regarding the range of installed capacity), the criterion of the voltage used in the network should be considered, namely, within a range not higher than that corresponding to medium voltages. Any connection to the national power system should therefore be at the level of the power distribution network (low voltage, seldom medium voltage). If a microgrid is to operate in island mode in the long term, it must meet high requirements in terms of having sufficient energy storage capacity or relying on high flexibility of internal energy demand. Consumers are open to participating in demand side response (DSR) programmes, offering their own energy usage flexibility in exchange for specific bonuses [42].

It should be noted that a microgrid itself cannot be considered part of the public distribution network. A microgrid does not exchange energy with the external network in a disorderly manner, like an inflexible prosumer, but in a properly controlled manner. From the network operator’s point of view, an intelligently managed microgrid can be treated as a flexumer [43]. Aggregator models that disregard the physical locations of generators and loads (e.g., virtual power plants) are not microgrids [44].

Microgrids can be hierarchically composed of smaller networks or installations with characteristics of autonomous management and, at least partial, self-balancing, and can also be combined into larger structures [45] (sharing energy, e.g., within a community [16]).

A microgrid operator is more than just an aggregator of small generators, a network service provider, a load controller or an emissions regulator—it performs all these functions and serves multiple economic, technical and environmental objectives. One of the main advantages of the microgrid concept over other ‘smart’ solutions is its ability to deal with the conflicting interests of different stakeholders in order to achieve a globally optimal operational decision for all players involved.

Microgrids are increasingly recognised as a game-changing solution to today’s energy challenges, driven by the need for efficient, resilient and sustainable energy systems. It is considered that the smart grid is an advanced form of the microgrid, enhanced by information and communication technology (ICT) to improve grid operations and customer service [15,46].

## 3. Types of Microgrids

Due to the lack of a universally recognised classification and their lesser popularity, ‘picogrid’, ‘nanogrid’, ‘minigrid’ and ‘miligrid’ will not be distinguished in the rest of this article, and all structures with the above-mentioned characteristics will be referred to as ‘microgrids’.

As technical facilities, energy microgrids can be categorised according to various criteria characterising their structure and resulting from their functionality, so there is a wide variety and multitude of practical solutions. Microgrids are used in various sectors, including residential areas, shopping centres, industrial facilities, and even transport hubs such as airports and ports [47] (Table 1).

One of the distinguishing features of a microgrid is its connection to the external power grid. A microgrid may comprise a single installation or a conglomerate of multiple facilities belonging to different entities (see Table 2).

Among the various types of microgrids, certain configurations demonstrate a significantly higher predisposition toward achieving energy self-sufficiency. The most prominent are off-grid (islanded) systems, which by design must ensure continuous, independent energy supply, often in the absence of any connection to the public grid. Such systems are commonly deployed in remote or inaccessible locations, including isolated settlements, research stations, or military installations, where autonomy is a necessity rather than a choice. Similarly, microgrids based on renewable energy sources (such as solar, wind, hydro or biomass), when supported by sufficient storage capacity and effective energy management, are inherently well-suited for self-sufficient operation. High levels of autonomy are also characteristic of mobile or temporary microgrids, which are intended to function independently in field conditions, for instance during emergency response operations or military deployments. Furthermore, distributed control architectures increase the resilience and local decision-making capability of microgrids, making them more robust against external disruptions and more conducive to autonomous operation. In contrast, grid-connected microgrids, particularly those operating in urban or industrial settings, generally prioritise energy efficiency, reliability, and cost optimisation over full independence, although many retain the capability to operate in island mode temporarily. Consequently, the degree of energy self-sufficiency is closely related to the microgrid’s operational mode, location, and strategic purpose.

## 4. Microgrid Components

Microgrids, as platforms integrating energy supply units (microgeneration), consumption units (controlled loads) and energy storage units within their own local distribution network, must have a structure management system in order to intelligently utilise the opportunities offered by the interaction of these components and achieve energy self-sufficiency for users. In modern civilisation, the most popular and useful form of energy is electricity, so a microgrid should primarily include electricity conversion and distribution systems. The basic components that make up an energy microgrid are discussed below.

### 4.1. Management System

Microgrids in the field of electricity distribution can use hybrid AC/DC networks to integrate multiple energy sources and energy storage systems. At the heart of this integration are energy management systems (EMS), which support control, energy management and the reliability of such an integrated system [55].

The management system does not operate in an isolated environment and cannot ignore a number of factors, which can be divided into [18,19,56,57,58].

- Internal, resulting from the technical conditions for the correct operation of the microgrid:
  ○ Energy level in storage facilities (e.g., batteries, heat storage tanks);
  ○ Quality requirements for the energy supplied;
  ○ Rules for operating the microgrid (e.g., voltage conditions, load limits on power lines, possibilities for regulating flows and reactive power, permissible parameters for the storage and transmission of other energy carriers).
- External, resulting from the environment in which the microgrid operates:
  ○ Availability of carriers, supply of primary energy resources (including weather conditions);
  ○ Requirements of the external distribution network operator (in the case of cooperation with the national power system).
- Resulting from the specific nature of the activity served by the microgrid:
  ○ Instantaneous power demand of consumers;
  ○ Optimisation of operating costs.

User requirements depend on the expected functionality of the facilities (e.g., resulting from production processes—for industrial microgrids, municipal and residential needs—for microgrids in buildings).

A comprehensive energy management solution incorporates process data from control and data acquisition systems, network monitoring, inverters, system automation, and other components. Additionally, the remote cards are designed as plug-and-play devices, allowing for fast and easy installation of the management system [53].

A microgrid requires an effective EMS to oversee the operation of its intricate network of electrical, thermal, and mechanical components. This system should deliver both short-term results—by adjusting to fluctuations in demand and production—and long-term benefits—by extending the lifespan of the most expensive and sensitive microgrid elements. Ultimately, this leads to lower system costs and greater overall benefits [59].

The technical and mechanical aspects of EMS must be carefully maintained to improve system reliability by effectively managing both user and utility sites. An optimisation technique is essential for EMS to regulate the actions of consumers, stakeholders, and utilities concerning production, consumption, market demand, and cost [15].

Microgrid optimisation aims to maximise the instantaneous power output of generators, extend the lifetime of the energy storage system (ESS), and minimise environmental impact and operating costs. To achieve this, it is necessary to define constraints and objective functions that address the microgrid’s operating costs.

A broader approach to decision-making by EMS includes, in addition to model-based methods (such as distributed optimisation based on consensus algorithms, game theory, and MPC (model predictive control) algorithms, data-driven methods whose main feature is the lack of a model of the system to perform energy management (e.g., stochastic, robust, distributionally robust optimisation algorithms, deep neural networks, reinforcement learning) [45].

For such a management system to work efficiently, it must be based on new technologies that enable multi-criteria optimisation in changing conditions, taking into account multiple parameters in real time. Methods and techniques based on AI, technologies using Blockchain and IoT (under the collective name BIoT) are used here. Various optimisation techniques are used [20,59]—Table 3:

The system has three main control models, each with its own advantages and disadvantages. Distributed control may be a good compromise, providing satisfactory quasi-optimal solutions at reasonable computational costs and communication layer complexity (see Table 4).

Centralised control is often used in scenarios where a high level of coordination is required, such as in grid-connected microgrids where the central controller can manage power flow and ensure stability [63,64]. Decentralised approach is suitable for islanded microgrids or scenarios where reliability and fault tolerance are critical, such as in remote or off-grid locations [65,66,67].

Distributed control is suitable for complex microgrid configurations, including networked microgrids and hybrid AC/DC microgrids, where coordination among multiple distributed energy resources (DERs) is necessary [68,69]. The distributed control scheme allows for a combination of centralised and decentralised control to a certain extent. In this approach, each local controller gathers information such as voltage and frequency from nearby units. This local data exchange, facilitated through two-way communication links, supports the central controller in achieving a global solution [62].

In simple terms, it can be said that centralised control is effective for small-scale microgrids, while decentralised control is better suited for large-scale microgrids. [16]. However, the choice of control strategy in microgrids depends on the specific application scenario, desired reliability, and system complexity. Centralised control is suitable for highly coordinated tasks, decentralised control excels in reliability and fault tolerance, and distributed control offers a balanced approach for complex and scalable systems.

An example of a central microgrid controller project, integrating metering, monitoring and energy management, is presented in [70]; it cooperates with, among other things, an energy demand forecasting module and an energy storage system. The paper [71] presents experience with the design and implementation of a microgrid in a commercial building, based on a centralised hierarchical control strategy to optimise its functionality. The implementation of the microgrid significantly reduced the electricity costs for connected loads in the building by 19.5%. Case studies comparison table for decentralised and distributed control strategies for microgrids can be found in [69].

Energy management objectives and strategies in a microgrid are shaped by user preferences. Because microgrids are modular and highly customizable, each one has distinct goals influenced by factors like geographic location, the types of installed equipment and loads, energy tariff structures, government policies, and the available energy storage and generation options within the microgrid [20].

An additional issue is emergency management strategies, which should be anticipated by the microgrid management system. These strategies are designed to increase the reliability of the microgrid in the event of potential random events, such as N-1 events, N-2 events, overloads, excessive generation, and short circuits. Emergency management strategies can be divided into preventive measures, protection against failures, and management of power outages [72].

### 4.2. Energy Sources

Another group of mandatory components of a microgrid structure are its own energy generation units, whereby a microgrid can be a multi-energy structure, i.e., in addition to electricity sources, it can also include sources of other forms of energy [73]. These sources are characterised by belonging to distributed generation sources with a single source capacity of less than 100 MW. The diversity of technologies promotes diversification, with zero-emission technologies being desirable, and modular SMR (Small Modular Reactors) are also being considered for use in microgrids [74].

Technologies used in a microgrid encompass both emerging and established solutions. These include innovative concepts like combined heat and power (CHP), micro wind turbines (MWTs), photovoltaic (PV) systems, microturbines, and fuel cells, alongside well-established technologies such as synchronous generators powered by DC engines, single-phase and three-phase induction generators, and small-scale hydroelectric systems. Dedicated chapters are devoted to the use of renewable sources in energy generation for microgrids, e.g., [75,76].

A special form of zero-emission energy generation in microgrids, which distributes heat in addition to electricity, is the recovery of thermal energy [77,78,79]. Heat recovery is an increasingly popular method in industry for reducing energy costs and minimising environmental impact. Heat recovery involves capturing and reusing heat generated during industrial processes, thereby reducing the amount of energy that would otherwise be wasted (Table 5).

### 4.3. Energy Storage

Energy storage systems are tools that facilitate the self-balancing of microgrids. The energy storage system is crucial in regions with unpredictable weather conditions because of its ability to store energy and maintain a balance between energy supply and demand [59].

Technologies enabling effective storage in the short term (between times of day or a maximum of several days) and in the longer term (between seasons) are used. Storage can take the form of electricity (electrochemical, electromagnetic field), heat or fuel (including hydrogen)—Table 6.

### 4.4. Controllable and Categorised Loads

The microgrid has to manage the loads by accomplishing the following tasks [91]:

- Load monitoring, analytics, and prediction.
- Load balancing and demand response.
- Load shedding for non-crucial loads to fulfil the net import or export power in on-grid mode.
- Stabilise the voltage and frequency in the islanded mode.
- Enhance the power quality and reliability of critical loads.
- Load scheduling and resource planning for the resilient operation.

Loads can be categorised into four types: noncontrollable, shiftable, controllable comfort-based, and controllable energy-based loads. Controllable loads, often referred to as smart loads, combine noncritical loads—capable of tolerating short-term voltage or frequency fluctuations—with a power electronic interface that electrically isolates the load from the power source [59].

The simplest classification of loads divides them into two categories: critical loads, which must operate to ensure the safety of microgrid users, and other (non-critical) loads—Table 7.

### 4.5. Microgrid Equipment to Support the Achievement of Energy Self-Sufficiency

Achieving energy self-sufficiency through a microgrid requires not only the presence of basic components such as energy sources, storage systems and management systems, but also their proper integration and ability to operate autonomously in dynamically changing conditions. It is crucial to equip microgrids with technologies that enable:

- independence from external energy suppliers
- full control of the local energy balance,
- flexible supply and demand management,
- dynamic adaptation to environmental conditions,
- use of locally available energy resources,
- response to emergency and crisis situations.

Self-sufficiency begins at the level of local, low-emission energy sources, whose operation is supported by systems that enable them to adapt flexibly to changing weather conditions. To this end, microgrids are equipped with AC/DC converters and multi-channel inverters, including grid-forming inverters, which allow different energy sources to interact in a hybrid AC/DC structure [92]. In addition, optimising production involves the use of control systems such as solar tracking systems and MPP (Maximum Power Point) controllers for PV systems, which enable real-time maximisation of solar panel efficiency [93], or automatic control systems for the direction and angle of microturbine blades.

Energy storage in a self-sustaining microgrid acts not only as a time buffer, but also as an element to stabilise grid parameters. For this purpose, it can be used [94,95,96]:

- Integrated Multi-Modal Storage Systems, combining, e.g., Li-Ion batteries with Thermal Energy Storage (TES) and hydrogen storage, so that energy can be balanced in different forms,
- Energy storage management systems (BMS/ESS Controller) interfacing with the EMS, allowing intelligent charging, discharging and reserving of resources depending on demand forecasts and scenarios,
- Inertial stabilisation resources (e.g., flywheels or supercapacitors) to provide rapid responses to voltage or frequency spikes, critical in islanding mode.

At the heart of the self-sustaining microgrid is the decision-making system, supported by an extensive measurement and control infrastructure. Key elements include:

- A network of energy sensors monitoring real-time generation, consumption, energy quality and equipment condition parameters
- Demand and production forecasting systems (e.g., based on machine learning), taking into account weather factors, equipment operating schedules and historical data,
- Local automation systems (PLC, HMI, SCADA), enabling instantaneous responses to system changes and control of resources without human intervention,
- Cybersecurity systems to protect user data and guarantee the integrity of EMS decisions.

Self-sufficiency requires not only ensuring energy supply, but also optimising consumption, coordinating the management of different types of energy resources in a given community to minimise energy costs [97]. To this end, the microgrid should be equipped with:

- Dynamic load management (DR/DSM) systems, allowing demand shifting and critical consumption reduction (load shedding),
- Managed loads with adaptive comfort functions—e.g., HVAC systems or lighting systems that react to occupant presence or energy availability,
- Intelligent terminal devices to communicate with the EMS and control energy consumption according to an optimisation algorithm,

A fundamental aspect of microgrid self-sufficiency is the ability to operate in island mode. This requires:

- Automation to separate the microgrid from the host network, while maintaining the stability of network parameters
- Mechanisms to automatically re-establish the connection (re-synchronisation) with the power system, while ensuring the technical conditions for synchronisation,
- Backup control logic and emergency systems, operating independently of the external network.

## 5. Sensors in Microgrids

### 5.1. Overview

Intelligent sensors are key to real-time monitoring and control of microgrid components, ensuring efficient and reliable operation [24,98]. Sensors enable coordinated and automated responses to changes in energy demand and supply [98,99]. They are used to collect and store data on energy production, consumption and storage, which is essential for predictive maintenance and optimisation of energy resources [98,100]. Thanks to the integration of smart sensors and advanced control systems, microgrids can significantly improve energy efficiency and reduce operating costs [101]. However, the huge amount of data generated by sensors requires robust data management and analytics to optimise energy management [100,102].

Various types of voltage and current sensors are installed in the smart grid to detect and identify system or line faults. With the support of Information and Communication Technology (ICT) and the Internet of Things (IoT), these sensors enable automatic fault recovery actions to be initiated promptly [15].

To maintain the reliability and safety of power transmission and distribution systems, various sensors are utilised. Current and voltage sensors are crucial for detecting faults, evaluating the performance of relays, and protecting the electrical grid. For example, non-contact inductive current sensors identify faults by detecting changes in current flow. Additionally, when high current loads cause transmission line temperatures to rise, surface acoustic temperature sensors [103] are used to monitor these increases, helping to prevent overcurrent issues.

Furthermore, issues like short circuits, wiring errors, or lightning strikes can cause power quality problems, leading to voltage and current fluctuations that could harm consumer devices. To address this, online sensors such as power quality monitors control the quality of the power supply, thereby contributing to reductions in power outages and load shedding. Fibre-optic sensors offer comprehensive monitoring of overhead transmission lines, tracking parameters like stress, strain, tension, magnetic fields, acceleration, and temperature. Finally, smart continuity grid sensors are employed to optimise electricity usage and enhance overall availability.

Smart Meters are equipped for real-time data collection, directly measuring key electrical parameters such as voltage, current, frequency, and phase angle. Thanks to their bidirectional communication, this recorded data is then securely and continuously transmitted to both the service provider and the customer. A crucial feature of Smart Meters is their ability to remotely manage the power supply-to-demand balance; they can connect or disconnect loads and control end-user power usage to help decrease overall consumption.

Current transformers and voltage transformers act as essential connections between measurement equipment—such as data acquisition systems and phasor measurement units—and the power system. However, harsh weather and challenging operating conditions can speed up transformer ageing, raising the likelihood of saturation. If transformer saturation is not properly managed, it can negatively impact the online monitoring, control, and protection mechanisms of microgrids [104,105].

Intelligent resource management involves responding to the effects of changing factors. Appropriately selected sensors are used to monitor these factors. The following types of sensors are used in microgrids, listed in order of popularity [24]: temperature, current, voltage, power, humidity, gas, frequency, pressure, vibration, and light. Examples of different types of sensors that can be used in smart microgrids are listed in Table 8.

Sensors play a crucial role in the operation and management of energy microgrids, contributing to their efficiency, reliability, and sustainability. Sensors enable the collection of real-time data on power characteristics, such as voltage, current, frequency, and power generation. This data is essential for monitoring and controlling the microgrid’s operations [174,175,176]. Advanced sensors, such as Waveform Measurement Units (WMUs), are used to detect disturbances and faults within the microgrid. These sensors provide GPS-synchronised measurements that help in quickly identifying and locating faults [177,178].

Sensors that monitor the condition of equipment, including partial discharge, infrared and vibration sensors, play a key role in maintaining the reliability and efficiency of microgrids. These sensors, combined with advanced communication and control systems, enable real-time monitoring and proactive maintenance, ensuring stable and secure microgrid operation.

Partial discharge (PD) measurement is a widely accepted method for assessing the health of electrical equipment, particularly for insulation systems in power components like generators, transformers, and switchgear [179,180]. PD sensors such as high-frequency E-field (D-dot) sensors, Rogowski coils, and loop antennas are effective due to their high signal-to-noise ratio and accuracy [179]. Low-cost RF detectors for PD monitoring provide a scalable solution for wide-scale deployment without extensive wiring, making them suitable for microgrids [181]. Infrared photoelectric sensors are used to detect temperature changes in electrical equipment, which can indicate insulation faults or excessive temperature rise [182]. Vibration sensors are crucial for monitoring mechanical conditions in microgrids. Sensors with AC outputs are preferred for detailed analysis using FFT (Fast Fourier Transform) to assess vibration amplitude and nature, while DC output sensors are used for machine protection, providing velocity or acceleration data [183]. Various sensors, including piezoelectric, electrodynamic, and magnetic sensors, are employed for vibration monitoring, each suited to specific applications and environmental conditions [184]. Supervisory Control and Data Acquisition (SCADA) systems are integrated into microgrids for centralised monitoring and control, using wired and wireless sensor networks to track vital parameters and operational status [185,186].

An energy management model for microgrids involves data acquisition systems, supervised control, human–machine interface (HMI), and monitoring and analysis of meteorological variable data [18]. Sensors facilitate the optimisation of energy resources by providing accurate data for energy management systems (EMS). This helps in balancing the energy supply and demand, ensuring stable and efficient operation [62,187]. The data collected by sensors can be logged and analysed to predict future energy needs and costs, allowing for better planning and utilisation of renewable energy sources [174]. A closed-loop mechanism in EMS involves continuous data collection, analysis, and feedback to optimise energy management. Sensor networks play a pivotal role by providing real-time data on energy consumption and production [188]. The intricate design of a microgrid system necessitates the use of various sensors and monitoring devices, which generate diverse types of data—including structured data from traditional power systems, semi-structured data such as images from cameras, and unstructured data from sources like weather information, network configurations, and maps [62].

The connection of a large number of small and technically diverse energy sources in a microgrid can lead to voltage regulation problems, which poses a challenge for EMS. The article [189] proposes several sensor selection schemes that aim to improve voltage measurement efficiency in microgrids, extend the service life of sensor networks, and ensure real-time communication between distributed sensors and an intelligent control centre.

The EMS, thanks to a system of wireless sensors designed to monitor various variables from renewable sources, can effectively conduct balance energy demand and production and support the efficient use of energy resources [24,190]. Modern EMS leverage big data and predictive analytics to enable real-time system control and actionable insights [191]. The integration of data from diverse sources, such as smart meters and wireless sensors, enhances the management of decentralised energy systems, including electrical storage and renewable energy generation [192].

A review of applied sensor, communication and monitoring technologies for microgrids with case studies, to demonstrate the importance of mutual knowledge awareness between the power grid domain and the communication network was made in [193].

Sensors are integral in managing distributed energy resources (DERs) such as solar panels and wind turbines. They help in monitoring the generation patterns and optimising the use of renewable energy [99,194,195]. The integration of Internet of Things (IoT) technologies with sensors enhances the real-time monitoring and control of renewable energy systems, improving the overall efficiency of the microgrid [196,197].

Wireless Sensor Networks enable the deployment of sensors without existing infrastructure, facilitating the efficient and reliable transport of data. This is crucial for the continuous monitoring and control of microgrid operations [198]. Sensors combined with cloud computing can handle large volumes of data, providing a scalable solution for data processing and control in microgrids [199]. Dedicated solar sources can also be used to power wireless sensor networks for intelligent monitoring of isolated microgrids for energy scheduling using simultaneous wireless transmission of information and energy, but the problem of optimising scheduling and optimising the placement of photovoltaic cells needs to be solved here [200].

By optimising the use of renewable energy and improving the efficiency of energy management, sensors help in reducing operational costs and electricity bills [187,201]. Effective sensor-based monitoring and control systems contribute to minimising carbon emissions by ensuring the optimal use of renewable energy sources [201].

Developing fault-tolerant control strategies for sensors is essential to maintain robust performance and enhance the stability of microgrids [178]. Continuous advancements in sensor technologies, such as self-powered sensors and multi-parameter sensors, are expected to further improve the efficiency and reliability of microgrids [202].

In the context of future sensor technology solutions dedicated to microgrids, it is worth taking a look at the latest technologies that are currently being considered. One example is quantum sensors, which can provide unmatched levels of sensitivity, precision and durability. Their use is being considered in areas of urban infrastructure, including energy [203], and therefore they have potential applications in microgrids. In turn, microgrids in which energy use is linked, for example, to the presence of users or specific facilities, can utilise modern flexible sensor technology [204,205]. The growing number of sensors also increases the demand for energy to power them, which will increase the load on the microgrid itself. However, there is a solution here too, namely the noteworthy technology of self-powered sensors [206,207].

### 5.2. Fundamental Value of Sensor Technology in Improving Microgrid Efficiency

Microgrids need to be able to collect, process and respond to real-time data in order to maintain stable, reliable and cost-effective operations. Sensors provide the necessary data-driven information to enable intelligent decision-making in several operational aspects, from power generation to demand management. The value of sensor technology in enhancing microgrid performance manifests itself in the following aspects:

- Real-time monitoring and control—sensors can detect voltage or frequency fluctuations, providing early warning signals for control systems that can react proactively to avoid disruptions; by monitoring energy distributions, they help the management system optimise decisions regarding energy distribution and storage; monitoring environmental variables (e.g., humidity, temperature), ensures that equipment performance is maintained within optimum ranges, preventing overheating or degradation of the stability and efficiency of equipment components.
- Predictive maintenance and fault detection—by continuously tracking parameters such as system temperature, vibration and electrical anomalies, sensors can identify early signs of wear or malfunction in electro-environmental equipment (distribution, storage, generation and transformation of energy carriers); this data can be analysed using advanced machine learning algorithms to predict failures before they occur, enabling pre-emptive action rather than reactive repair, reducing downtime and extending the life of assets, resulting in cost savings and increased overall system efficiency.
- Demand response and load optimisation—in microgrids, managing energy consumption and matching it to supply is key to maintaining self-sufficiency and ensuring that resources are not over- or under-utilised; sensors in smart meters and IoT-enabled devices (sensors of energy parameters and parameters affecting energy consumption) allow demand management by providing real-time data on the energy consumption of homes or businesses and transmitting it to a smart energy management system (EMS) to optimise energy allocation and avoid overloading the microgrid, also reducing consumption during peak hours or shifting the load to periods with excess renewable energy.
- Renewable energy integration and forecasting—the predictive capability of sensor systems helps improve the integration of different energy resources. Sensors track weather patterns and environmental factors, providing accurate predictions of renewable energy generation. By integrating this data with energy management systems, microgrids can better predict the availability of renewable energy and adjust storage or consumption accordingly. Advanced algorithms, using sensor data, can further predict the output of renewable energy sources and adjust storage management strategies, increasing microgrid efficiency.
- Fault tolerance and grid resilience—sensor information enables rapid fault detection and self-repair of the microgrid; sensors can quickly identify the location of a fault and contribute to isolating it; sensors in the control system can provide signals to regulate network voltage and frequency in response to external disturbances or load changes. With a high-density sensor network across the microgrid, the system can automatically reconfigure itself to bypass faulty areas or sources of failure, thereby improving both resilience and performance, even under extreme conditions.

### 5.3. The Role of Sensors in Achieving Energy Self-Sufficiency in Microgrids

The use of sensor technology in microgrids not only improves their operational efficiency, but is also a key factor in achieving energy self-sufficiency. This involves the appropriate integration of various sensors into the smart grid structure in order to create intelligent security strategies capable of processing and analysing large amounts of data, which facilitates real-time decision-making and precise fault detection [208]. Sensors form the basis of this capability through the following mechanisms:

- Balancing local energy production and consumption—sensors enable microgrids to continuously measure in real time both supply (from PV systems, wind turbines, CHP, etc.) and energy demand (homes, industrial facilities, critical infrastructure) [209]. This data allows dynamic adjustment of microgrid operation and ensures that the energy produced is used locally with minimal losses.
- Real-time energy management—by integrating sensor data with EMS and SCADA systems [210], microgrids can automatically adjust the operation of energy sources, energy storage and loads, using the potential of users/flexumers.
- Efficient use of energy storage resources—sensors monitoring voltage levels and other operating parameters enable intelligent management of the charging and discharging cycles. Maintaining storage units in optimum operating conditions extends their lifespan and allows them to efficiently collect surplus energy from RES. [211].
- Autonomous response to disturbances—voltage, current, frequency and power quality sensors enable rapid fault detection and initiation of corrective action without the need for external intervention. In this way, the microgrid can autonomously maintain stable operation under fault conditions or disconnection from the master network. Synchronised sensor technologies help solve the event location identification problem, for different types of steady-state and transient events [212].
- Autonomous monitoring of microgrid operating parameters—measurement of electrical quantities (e.g., as part of smart metering) at selected locations, enabling estimation of operating conditions, maintenance of reliability and safety of energy use, minimisation of losses and location of faults [213].

## 6. Microgrids in a Transforming Energy Sector

### 6.1. Decarbonisation

Decarbonisation of the economy is a global trend, especially in developed countries, due to the inclusion of carbon footprints in economic calculations and the possibility of gaining a competitive advantage in ‘green’ investments. Full decarbonisation is possible, but difficult in terms of full technical and organisational implementation. In this respect, microgrids can be a tool for decarbonisation, as a place for the application of zero-emission technologies, the electrification of various branches of the economy and means of production. Optimisation in microgrids through EMS promotes energy efficiency by reducing the demand for energy from non-renewable sources, leveraging energy storage and load management.

In the context of the ongoing development of microgrids and their anticipated role in future power grids, the plan for developing new solutions can be divided into three closely related areas: (1) integration of energy storage systems (EES) in microgrids and power plants, (2) active demand management, and (3) improvement of microgrid control and monitoring capabilities [126]. All these tasks can be carried out in such a way that energy based on microgrid structures does not depend on fossil fuels.

Microgrids play a positive role in the decarbonisation of the energy sector, enabling greater penetration of renewable energy sources and reducing dependence on fossil fuels [214]. The use of renewable energy sources in microgrids contributes to the reduction in greenhouse gas emissions and supports global sustainable development goals [215]. For example, the microgrid project for KENTECH in South Korea aims to achieve up to 100% self-sufficiency and a 60% reduction in CO_2_ emissions [216]. Simulations of microgrid operation confirm the effectiveness of such a structure as a tool for reducing CO_2_ emissions [217,218].

Advanced energy storage solutions help capture and utilise energy during peak production periods, ensuring a reliable power supply even when renewable energy sources are unstable [219].

Microgrids are composed of different distributed energy resources, which are operated differently from individual sources. Studies presented in the literature, e.g., [220] shows the theoretical maximum benefits of microgrids (with own renewable energy sources) in energy efficiency improvement and GHG emissions reduction compared to conventional energy supply systems.

Support for decarbonisation through electrification within an industrial microgrid may consist, in particular, of:

- Using heat pumps instead of fossil fuels to produce low- and medium-temperature indirect heat.
- Using electrolysers to produce hydrogen from water electrolysis instead of natural gas.
- Electrification of transport within the area covered by the microgrid.

Another goal of decarbonisation is related to sustainable development and improving competitiveness. Here, too, EMS tools come into play. This involves intelligent management of zero-emission resources, optimisation to improve energy efficiency, and the use of price arbitrage to reduce energy costs. Own generation near consumption points means independence from energy supplies from the power system and external commodity markets, as well as reduced energy transmission losses. All this contributes to lower operating costs and thus improves competitiveness. In addition, thanks to the flexibility of energy resources integrated into microgrids, it is possible to provide additional services to the power grid and generate income (Table 9).

In the context of energy transition, it is worth noting the characteristics of a hybrid microgrid, namely that thanks to control systems and EMS, such a structure does not operate chaotically, but thanks to adapted inverters, especially GFM (Grid Forming Inverter) type, which simulate the operation of a physical synchronous machine and, in cooperation with energy storage, can become a reservoir of artificial inertia for the power system.

Furthermore, generators used in microgrids, operating in cogeneration and biogas systems, are usually synchronous machines, i.e., they provide inertia [221]. These systems are also sources of short-circuit power, which translates into grid resilience. Therefore, a properly constructed and managed microgrid has the potential to support the power system in its normal operation and in emergency situations. Inertia resources are particularly desirable in systems with high RES generation (mainly PV), where traditional units based on synchronous generators, connected directly to the grid, are being replaced by units connected to the grid via inverter systems.

This issue is related to the problem of changing the operating paradigm of electricity network operators, both distribution and transmission. In a structure consisting of autonomous microgrids connected by distribution lines, the operator will be a neutral, physical aggregator of these microgrids, maintaining system traffic within a given area. The operator will manage the energy exchange platform between prosumers or microgrids [222]. One of the major advantages interconnected microgrids offer is the distributed structure, which improves the reliability of the distribution network [223].

### 6.2. Integration of Different Energy Technologies

#### 6.2.1. Overview

A microgrid can combine different energy technologies and can be considered at various stages of energy transformation—from generation through distribution to the obvious end use of energy. In terms of electricity, both AC and DC distribution technologies can be used within it. Integration can also involve combining different energy carriers, including within polygeneration.

The integration of different energy technologies in microgrids refers to the structured incorporation of multiple technologies into a single, coordinated system that can operate both in connection with the main grid and independently. Despite its limited size, the structure can combine multiple technical and organisational solutions. Technology integration can take place at the level of energy generation, storage, distribution and conversion, as well as billing for usage [224]. These technologies are coordinated to optimise the efficiency, reliability and sustainability of the local energy supply system. This integration is facilitated by advanced power converters and energy storage devices, which increase the efficiency and reliability of microgrids [225,226]. In a microgrid that interconnects various power generation systems utilising different technologies and power capacities, it is essential to adopt a hierarchical control framework aimed at reducing operational costs while enhancing efficiency, reliability, and controllability [126].

Microgrids facilitate the integration of renewable energy sources such as solar, wind and biomass, which are essential for reducing carbon dioxide emissions and achieving sustainable development goals [47,76]. For example, a typical microgrid may use photovoltaics and wind turbines for primary renewable energy generation, diesel generators for backup, batteries for energy smoothing and peak load reduction [227], and Vehicle-to-Grid (V2G) systems to handle bidirectional energy flows as an additional form of storage [228]. In addition to battery management, hydrogen fuel cells are used as long-term, clean, backup power sources. Batteries and hydrogen storage facilities help manage the instability of renewable energy supplies and ensure a stable power supply [229,230]. The aim is to ensure stable, resilient and low-carbon local energy systems that can adapt to changing load requirements and generation profiles [1,88,231].

Active cooperation with the network or island operation requires flexibility in both generation units and consumption. Such a structure requires intelligent management; hence, the microgrid management system is a crucial component [53]. Hybrid AC/DC architectures enable simultaneous operation of AC and DC systems, facilitating the effective integration of a wider range of technologies [55]. Networked or clustered microgrids enable energy sharing between multiple microgrids, further increasing reliability and enabling greater participation of renewable energy sources [232].

Intelligent management of an extensive microgrid, collecting data from multiple sensors, and the operation of a management system that maintains energy balance in conditions of variable consumption and generation from renewable energy sources requires real-time data analysis. Edge computing, i.e., a model of local data processing on end devices, network gateways or microservers, can be used here, which significantly reduces delays [233]. Edge computing assists in real-time energy management, load balancing, and proactive maintenance of renewable energy sources like solar panels and wind turbines [234]. In [235], a microgrid-oriented architecture was proposed, including edge-cloud collaboration for data processing, network communication, and security mechanisms, demonstrated in a rural community in Central China.

The development of advanced control strategies, including artificial intelligence (AI) and machine learning (ML), is crucial for optimising the operation of microgrids. These technologies enable real-time monitoring, predictive maintenance and adaptive control, thereby improving the stability and resilience of the network [236]. The increasing digitisation of building management and the transition to smart grids are improving the assessment and optimisation of energy efficiency in real time in microgrids [237,238]. New technologies such as artificial intelligence and quantum computers are expected to further optimise energy management and increase the capabilities of microgrids [239]. Artificial intelligence and machine learning are increasingly being used for energy management, improving the efficiency and reliability of microgrids by predicting energy demand and optimising resource allocation [240]. Modern microgrids use smart grid technologies, peer-to-peer energy markets, and advanced energy management systems to optimise efficiency and effectively integrate different energy sources [47,241].

An analysis of the operation of microgrids using artificial intelligence, including an analysis of bottlenecks and trends in the development of artificial intelligence, together with a proposal for an artificial intelligence application system enabling the smart microgrids, the main element of which is a digital twin, is presented in [242].

AI-based control strategies, such as adaptive learning and distributed control architectures, increase the resilience and sustainability of microgrids. These strategies support predictive energy management and intelligent decision-making processes. A review of recent research [243] highlights the effectiveness of artificial intelligence in overcoming key challenges related to the operation and management of microgrids. These include distributed control strategies, intelligent decision-making processes supported by advanced algorithms, and communication frameworks. The current state of AI use in microgrid management, based on an analysis of 187 relevant articles in the Scopus database, indicated that India, China and the United States have made the greatest contribution to research in this area [244]. Adaptive systems and optimisation algorithms are key research topics in this field.

An example of the implementation of a proactive energy management system in a microgrid in tribal settlements in India, with IoT components providing real-time data on energy production and consumption and using AI techniques, to forecast weather conditions, is described in [245]. The AI-machine learning-based management system controls resources between solar and biodiesel systems, with surplus energy stored through hydrogen production by electrolysis. The design has enabled a reduction in CO_2_ emissions of 11,680 kg and savings of 46 tonnes of carbon per year.

Based on a review of 200 scientific papers, research and a review of solutions for the implementation and optimisation of microgrid energy management systems (EMS) using artificial intelligence (AI) were presented in [246]. The research highlights the importance of hybrid systems, demand management and energy storage in addressing the instability of renewable energy sources, which will improve the sustainability of microgrids. The importance of deep learning methods for load forecasting and reinforcement learning for control optimisation has been emphasised.

The authors of the paper [247] analyse the role of AI in various areas of microgrids, such as design, where the emphasis is on optimal scaling; control, where a hierarchical structure is used; and maintenance, including condition monitoring, diagnostics and forecasting. The article also highlights the integration of the Internet of Things (IoT) to improve connectivity and data exchange, and blockchain technology to enhance cybersecurity.

The concept of blockchain in the energy sector is gaining popularity, offering new opportunities for secure and efficient energy transactions, including within microgrids [248,249,250,251]. The development of renewable distributed sources and microgrids requires a secure and transparent transaction system in which blockchain technology enables real-time peer-to-peer (P2P) exchanges between users without intermediaries. However, this carries the risk of attacks (so-called 51% attacks), in which a single entity can gain majority control over the network and manipulate transactions, energy production and consumption data, and even lead to instability in energy supply at the community level [252]. The paper [253] proposes the integration of a hybrid Proof of Work_Proof of Stake (PoW/PoS) consensus mechanism with a Long Short-Term Memory (LSTM) model to detect anomalies in blockchain-based microgrids in order to address issues in blockchain systems such as 51% attacks, double spending and high energy consumption, by optimising energy consumption and increasing security.

In the context of applying blockchain technology to peer-to-peer energy trading within microgrids, the article [254] discusses the necessary actions and implementation efforts in the areas of pricing mechanisms, privacy constraints, scalability, and the requirements of an overarching energy management system. The feasibility of the market mechanism and settlement process for direct electricity transactions between distributed generation units and consumers based on blockchain technology in the specific case of microgrids was presented in [255]. The issue of generation distribution and prosumption is linked to the problem of effective financial settlements between users within cooperating structures such as microgrids. Dedicated cryptocurrencies linked to renewable energy flows may prove to be an innovative solution [224].

Practical challenges related to the integration of technology within microgrids include [13]:

- Power quality, voltage, and frequency must be maintained within defined thresholds.
- Intermittent DERs may not provide consistent output, requiring more energy storage devices, which in turn demand additional space and frequent maintenance.
- Reconnecting and coordinating the microgrid with the main grid after a fault is a significant technical hurdle.
- Developing a reliable protection system poses a major engineering challenge.
- Microgrid performance can be negatively affected by net metering and idle costs.
- Appropriate standards for interconnection need to be developed.

Depending on the degree of development of the microgrid, various telecommunications technologies related to the transmission of measurement data and control signals can be used and integrated within its structure. The selection of communication technologies for microgrids in remote, residential, and rural regions is primarily determined by deployment costs and data transmission rates. Technologies such as WiFi, Bluetooth, Z-Wave, and Zigbee are commonly employed in these settings. Additionally, passive optical networks, along with 4G or 5G technologies, are utilised in microgrids serving public utilities [20].

The sensor nodes can utilise short-range wireless communication for information transmission. A Zigbee self-network is capable of performing data acquisition, storage, and communication in complex environments. The GPRS network facilitates data transmission between the aggregation node and the data monitoring centre. The microgrids monitoring centre is equipped with a database and microprocessor to store all collected data, analyse it, process the information, and support decision-making [256].

#### 6.2.2. Analytical Perspectives

In the context of microgrid applications, the integration of sensor technologies, optimisation algorithms, and energy storage systems (ESS) is crucial for ensuring efficiency, resilience, and sustainability. To achieve these objectives, it is essential to employ analytical perspectives that focus on monitoring, controlling, and optimising the interactions between these components in real-time.

Contemporary sensor portfolios span tightly time-synchronised phasor measurements (μPMUs), high-resolution inverter telemetry, smart meters and breaker/relay status, and an array of asset-level sensors that monitor cell voltage, temperature, and other health indicators. Exogenous sensing—irradiance, wind, ambient temperature, EV arrival events, and market signals—completes the picture required for predictive control.

Classical topology-aware placement techniques should be augmented with control-aware value-of-information: sensors placed not merely to minimise state estimator variance, but to maximise expected closed-loop performance (reduced constraint violation probability, lower energy cost, or reduced battery ageing). Time synchronisation, streaming quality checks, and compression/error-bounded transport protocols are essential to preserve the information content that advanced state estimators and controllers expect. Sensors should be placed where closed-loop performance sensitivity is highest. For example, installing μPMUs at nodes whose voltage or phase angle uncertainty has the largest effect on constraint violations or reserve activation accuracy maximises the operational return on sensing investment.

At the algorithmic level, state and parameter estimation must fuse heterogeneous streams under nontrivial physics. Anomaly classification—distinguishing sags, transients, cyber intrusions, and equipment faults—benefits from waveform analytics and model-based residuals that can be used to switch control modes (for instance, invoking conservative reserves or islanding) when trust in telemetry drops.

Real-time twins calibrated with live telemetry enable rapid validation of control policies, including injection of sensor faults and cyberattacks to test graceful degradation strategies. Twins also allow surrogate model validation and derivation of trust regions where lower-order models are acceptable.

The energy storage systems (ESS) portfolio for microgrids is diverse: Li-ion variants (LFP, NMC, LTO) offer high power density and fast response; flow batteries and hydrogen enable long-duration storage; supercapacitors and flywheels address sub-second stabilisation. Inverters can operate in grid-forming or grid-following modes, and ESS play roles ranging from synthetic inertia and fast frequency response to arbitrage and black-start capability. Hybrid ESS (HESS)—combining fast, low-energy devices (supercaps) with slower, high-energy batteries—is an effective architectural pattern. Optimal power/energy splitting between components is framed within joint model predictive control formulations that explicitly minimise a weighted sum of operational cost, service shortfall risk, and degradation. Co-sizing problems, solved in planning stages, are multi-objective: capex, life-cycle cost, and emissions must all be balanced.

Optimisation for resilience is mixed integer in nature (sequence scheduling, feeder energisation order) but can be relaxed into tractable approximations or solved offline to produce fast, precomputed plans for emergency use. Rigorous evaluation must report operational, economic, asset, and cyber-physical key performance indicators (KPIs): energy not supplied, frequency/voltage violation rates, reserve activation accuracy, total cost (including degradation), battery life consumption, thermal excursions, and detection delays/false positive rates for sensor anomalies. Comparative ablations—with and without μPMUs, with centralised versus distributed optimisation, with and without degradation-aware dispatch—reveal the true value of the integration. Hardware-in-the-loop tests and digital-twin stress scenarios (sensor dropout, spoofing, high-renewable ramps) should be standard practice prior to field rollout.

High-quality, time-synchronised sensing unlocks the observability required for advanced estimators; those estimators enable model predictive and robust optimisation that properly account for uncertainty and asset degradation; and these optimisation decisions realise the operational value of ESS while preserving their life. The engineering objective is therefore holistic co-design: place and secure the measurements that matter, choose models and algorithms with the right fidelity for each time scale, and embed degradation and resilience explicitly in dispatch.

### 6.3. The Question of Energy Self-Sufficiency Through Microgrids

#### 6.3.1. Comprehensive Framework

Microgrids play a key role in achieving energy self-sufficiency, increasing resilience and promoting environmental sustainability. By utilising local renewable energy sources and advanced energy management systems, microgrids can provide reliable and cost-effective energy solutions tailored to the specific needs of areas and regions [52,216,219]. These features together provide microgrid users with reliable and efficient energy supplies, making them virtually self-sufficient in terms of energy [59,257,258].

The issue of energy self-sufficiency through microgrids can be considered on two levels: systemic and local-individual. The individual level is related to the supply of energy to consumers within the microgrid’s area of operation from its own local sources and energy storage facilities. The system level is related to the possibility of utilising the potential of intelligently controlled microgrids in terms of their flexibility and generation from various sources to support the functioning of the system to which the microgrid is connected.

The main advantage of microgrids is their ability to generate and consume energy locally, which reduces the need for long-distance electricity transmission and increases energy efficiency [257,259]. This local generation capacity is crucial for maintaining a high level of self-sufficiency, especially in remote or isolated areas [258,260]. By generating and storing energy locally, microgrids can reduce energy costs and provide economic benefits to communities and businesses [52,261,262].

Microgrids can achieve a high level of self-sufficiency by using local renewable energy sources. For example, in the Netherlands, microgrids can operate self-sufficiently for 58% of their operating hours using photovoltaic panels, generators and batteries [263].

Microgrids can operate independently of the main grid (island mode) during power outages or crises, increasing the resilience and reliability of local energy supplies [52,227]. This option is particularly beneficial for remote or rural areas with limited access to centralised energy networks [219,264], and in areas with complex energy needs [265], including those adapted to the specific characteristics of diverse urban areas [239]. Furthermore, microgrids are emerging as a viable solution for rural electrification, providing sustainable and reliable access to energy for remotely located communities [264,266,267,268]. Microgrids are designed to provide reliable power during extreme weather events and other disruptions, making them a key component of resilient energy infrastructure [269].

A microgrid performs four core functions [72]:

- Information interaction—involving energy data analysis, privacy preservation, system state estimation, alarm and situational awareness, and forecasting of load and generation.
- Control and scheduling—covering uncertainty management, plug-and-play device integration, coordination of multiple energy sources, transitions between grid-connected and islanded modes, voltage and frequency control, and scheduling optimisation.
- Resilient operation—including proactive responses, emergency handling, system recovery, relay protection coordination, and cybersecurity measures.
- Ancillary services—which encompass market participation, demand response, congestion relief, spinning reserve provision, black start capabilities, and seamless integration with the main grid.

Specifically, ancillary services that can be provided by microgrids can be divided into the following categories, according to the operation modes of microgrids [44,126]:

- Grid-connected operation:
  ○ Frequency control support.
  ○ Voltage control support.
  ○ Congestion management.
  ○ Inertia emulation.
  ○ Power oscillation damping.
  ○ Unbalance compensation.
  ○ Reduction in grid losses.
  ○ Improvement of power quality (voltage dips, flicker, compensation of harmonics).
- Islanded operation:
  ○ Black start.
  ○ Grid-forming operation:
    − Frequency control.
    − Voltage control.

Microgrid users have the potential to participate in demand response programmes, optimising energy consumption and contributing to grid stability by adjusting consumption based on supply conditions [261,270].

Microgrid design involves complex configurations due to project-specific parameters such as stakeholder needs, resource availability, and existing infrastructure. Optimisation methodologies such as simulated annealing and particle swarm optimisation can help increase the degree of self-sufficiency [259,271]. The variability of renewable energy sources continues to pose a challenge to maintaining stable energy supplies. Advanced energy storage technologies and smart energy management systems are key to solving this problem [219,272].

Microgrids can optimise energy costs through effective energy management systems and demand response strategies. These systems help balance generation and consumption profiles, reducing net running costs and increasing economic benefits [273,274,275]. At the current stage of development, ensuring the economic viability of a self-sufficient microgrid requires careful consideration of investment costs, payback periods and incentive amounts. This period depends on individual circumstances, but due to technological developments and availability, payback periods are becoming shorter. For example, in Sivas, Turkey, a multi-carrier microgrid system using wind, solar and biomass energy has shown a payback period of less than 6.5 years [276].

#### 6.3.2. Conceptual Framework

To further analyse the issue, a framework for the self-sustainability and resilience of microgrids and the integrated optimisation of their performance is proposed below.

Self-sufficiency of microgrids goes beyond local energy generation and storage, it also requires a comprehensive approach that includes energy flexibility, storage management and interaction with the grid. To structure this understanding of the problem, we propose a framework for optimising microgrid self-sufficiency along the following dimensions:

1. Dynamic sizing and location, optimal deployment and technology selection of renewable sources and energy storage. This can be achieved through advanced optimisation techniques based on computational intelligence.
2. Generation and load prediction and forecasting. Techniques based on machine learning models can be used to predict daily and seasonal changes in renewable energy generation and user energy use preferences.
3. Integration with smart grids. Utilising the Internet of Things (IoT) and machine learning techniques, microgrids should be equipped with adaptive controllers that can dynamically adjust energy flows based on the current state of the system, external weather conditions and grid demand.
4. Edge Computing for Real-Time Decision-Making: In cases where microgrids operate autonomously (especially during islanded modes), edge computing can enable real-time data processing and quick decision-making without relying on distant cloud-based servers, reducing latency.
5. Blockchain for secure energy trading. To maximise economic viability, microgrids can integrate with local energy markets or demand response programmes. Blockchain technology can be used to provide secure, transparent and decentralised trade of surplus energy between consumers and the grid or other microgrids.
6. Collaborate with other microgrids and prosumers not directly physically interconnected but connected to the national electricity system (on a VPP—virtual power plant—basis). The VPP can aggregate microgrid resources and create a flexible, dispatchable energy pool, improving the self-sufficiency of the area, while allowing participation in wider energy markets.

Microgrids provide resilience by facilitating local generation during power outages, but resilience also depends on how quickly and efficiently a microgrid can recover from a disruption (e.g., an extreme weather event). Based on existing microgrid functions, we propose the following framework for microgrid resilience.

1. Predictive maintenance and failure detection. With IoT sensors and real-time analytics, microgrids can adopt predictive maintenance strategies to identify system failures before they occur. Techniques such as predictive analytics and digital twins (virtual microgrid models) can enable accurate failure prediction and resource allocation.
2. Optimising distributed energy resources (DER). Optimisation algorithms can ensure that microgrids have sufficient energy resources from complementary stable, local DERs to quickly restore power in islanding mode. These DERs can include distributed storage units, such as battery systems, combined heat and power (CHP) units or fuel cells, which are critical during extended outages.
3. Grid-Forming Inverters. To improve resilience, microgrids can incorporate grid-forming inverters, allowing the system to initiate its own power generation and manage frequency/voltage stability without external support.
4. Self-Healing Networks. A “self-healing” microgrid uses automation and intelligent controls to restore power automatically following a disruption. This could involve self-healing algorithms, which prioritise the restoration of critical loads while maintaining the stability of the system.
5. Dual-Mode Operation (Grid-Connected and Islanded). For hybrid operation, a framework should be developed to facilitate smooth transitions between grid-connected and islanded modes. This requires the ability to share ancillary services with the main grid, such as frequency regulation, voltage support, and power quality enhancement, while maintaining independence in critical times

## 7. Discussion

In relation to the gradually gaining popularity of small, autonomous and self-balancing energy networks, the general term ‘microgrids’ is used. The common meaning of this term does not include a specific criterion for the installed capacity of such a network. The term microgrid is usually limited to a restricted area of operation, covering at most a selected campus, building complex, park, settlement or housing estate.

There is no generally accepted classification criterion for the less commonly used terms nano-, pico- and mini-grids, and at the current stage of development of such structures, it does not seem necessary to distinguish between them. The term microgrids, which has gained the highest popularity, seems to be sufficient as a common and only inclusive term for all such structures for use in the literature. The criteria for belonging to nano-, pico-, micro-, mili-and mini-grids, particularly in terms of power capacity, should take into account the conditions and specific characteristics of the energy sector in a given country (different conditions for local energy use due, among other things, to a society’s wealth and level of development or climatic conditions), so they cannot be absolute and precise, which may lead to misunderstandings. This approach should therefore not be the basis for international standardisation. These misunderstandings can hinder understanding at the stage of describing and analysing phenomena in these structures. They may also translate into a design and technology selection process that is dependent on quantitative parameters (including capacity), leading to the selection of standardised components that prove to be size-unsuitable for the actual scope of local microgrids. In such a case, the design and component selection process should be individualised each time. However, with the development of technology and the ubiquity of prosumer solutions, standardised standards will be needed for the design and operation of the distinguishable structures now collectively referred to as microgrids. Classification of these structures should be based on functionality rather than size. Potential directions requiring standardisation of microgrids are signalled in Table 10.

Within the IEC organisation, issues of standardisation of smart electronic devices in power systems, including data models for microgrids and distributed energy sources, are dealt with by a working group in Technical Committee TC57, as well as by the IEC System Committee Smart Energy providing guidelines for the planning and design of microgrids, as well as their energy management systems. In the IEEE organisation, microgrid issues are addressed by working groups within the Power and Energy Society (PES). The extent of microgrid standardisation is and should be market-driven. It can be foreseen that attention will be paid to open microgrid structures, plug & play functionality and the ability to seamlessly combine RES and energy storage systems into an autonomous network. The complexity, diverse structures and lack of standardisation of newly developed microgrid structures make such deployments complex and costly, and thus furthermore dependent on state subsidies.

At the current stage of transformation and development of engineering practice, the single term ‘microgrid’ fully reflects the idea of these structures. The general definitions in the standards cited in Section 2 appear to be consistent, which should not lead to any serious misunderstandings in the designation of the concept. Microgrids are small, local energy systems that can operate independently (island mode) or in connection with the main grid (grid-connected mode). This dual functionality allows them to ensure continuity of supply even during power outages from the main grid, increasing reliability and energy resilience [52,88,260].

The growing interest in microgrid structures stems from the increasing popularity of distributed generation structures, which are more resilient to situations that could immobilise a generation unit (disasters, terrorist attacks and war). A blackout over a larger area is much more likely when energy is supplied from a single direction, from a few large sources. In the case of microgrids, the operating philosophy is different. Many smaller sources using different technologies can back each other up and supplement each other, with energy storage providing additional value in the form of stabilising the energy supply. Different microgrids can be connected to each other via a cyber-physical energy structure (networked microgrids). The linkage between microgrids can either restrict certain unfavourable characteristics or enable mutual support [45], enriching the national power system and, as such, be treated as an additional component of the transforming system, increasing the reliability and quality of power supply. Analyses carried out in connection with the war situation in Ukraine, where the national energy infrastructure is being brutally attacked, indicate the legitimacy and necessity of developing and deploying microgrid structures [277,278].

The development and spread of microgrid structures will require a paradigm shift in the functioning of network operators. They will transition from being monopolists to neutral infrastructure providers and digital operators of a new distribution system between microgrids. The system managed by such an operator will change from a vertical pyramid to a horizontal network of connections between active and flexible users. The tasks of the operator of such a system may include, in particular:

- Connection management
- Flexibility management (supervision of service provision by flexumers)
- Enabling energy exchange between users—prosumers and flexumers
- Providing information on the state of the network, load forecasting
- Resource coordination, area balancing, and maintaining power quality parameters
- Cooperation with other network operators.

The intelligence of an object, such as an energy system, means its ability to respond to changing factors. In order to determine whether such factors have occurred, devices are needed to record or measure characteristic values, i.e., the foundation of a properly functioning microgrid within a smart grid is appropriately selected and distributed sensors. This involves the use of electrical and non-electrical sensor technologies, working in conjunction with optimisation, estimation and prediction tools that use advanced AI methods.

In a new energy microgrid environment, sensor nodes can be categorised based on the type of information they collect into fixed nodes and mobile nodes. Fixed nodes are responsible for gathering data from specific devices within the microgrid, while mobile nodes are utilised to ensure the safety of on-site operations. Wireless charging technology, derived from wireless power transmission principles, uses electromagnetic resonance or electromagnetic coupling to transfer electrical energy from the power source to electrical equipment without the need for physical connections, enabling convenient energy transfer.

Sensors play a vital role in the functioning of energy microgrids by delivering real-time data essential for monitoring, controlling, optimising, and integrating renewable energy sources. They are key to enhancing the economic and environmental performance of microgrids, and continued advancements in sensor technology are expected to drive even further progress.

As smart grid solutions, including microgrids as specific components, develop and grow in popularity, the demand for various types of sensors will increase. A microgrid management system that meets user objectives, which boil down to reliable energy supply at the lowest possible cost while maintaining basic user comfort and/or maximising profits, must be based on reliable, up-to-date data and information that influences decisions regarding the control of microgrid components. As energy is a fundamental factor in activity and a means of production [279], the stage of managing it becomes particularly important and must take into account diverse technologies and methods [280].

Contemporary microgrids, striving to achieve full energy self-sufficiency, cannot limit themselves to implementing sensors as passive monitoring instruments. The data generated by these sensors is important—its quality, reliability and usefulness in decision-making processes. A key aspect of the functioning of modern microgrids is the ability to quickly and reliably acquire, transmit and process information in real time. This requires not only a properly designed communication infrastructure, but also intelligent analytical systems capable of dynamically responding to changes in energy demand, production or emergency situations. Data thus becomes the basis for microgrid autonomy, enabling optimised energy resource management, predictive infrastructure maintenance and adaptation to changing environmental and operational conditions. In this context, the role of sensors goes beyond mere measurement—they become an integral part of the decision-making system, supporting the continuity of operation, energy efficiency and operational independence of the microgrid.

The way energy is used is influenced by an increasing number of factors, which should be measured objectively, and this is where sensor systems play a huge role. Sensors in such an extensive structure can participate in the verification of assumptions and predictions (made using AI methods) of factors affecting energy demand. These factors can range from behavioural and social, through weather and climate, and economic, to strictly technical, related to the condition and capabilities of the available infrastructure. Smart energy, based on microgrids, will constitute a huge segment of applications for sensor technology in the near future. This will enable the achievement of a reasonable level of energy self-sufficiency at the local level.

Energy microgrids, being systemic solutions for combining energy consumption with on-site generation that can be integrated into the professional energy grid, can become one of the foundations for increasing energy security. Other benefits that can be seen in connection with the operation of microgrids can be considered on many levels—Table 11.

Implementing energy microgrids involves navigating a complex landscape of technical, economic, political, and social (including user acceptance) challenges—Table 12. While microgrids hold considerable promise for advancing energy resilience and sustainability, overcoming these challenges requires coordinated efforts across sectors, including innovation in energy technologies, supportive government policies, and active engagement with local communities. Addressing these challenges head-on will pave the way for the successful deployment of microgrids as a cornerstone of the future energy landscape.

Addressing these challenges requires innovative solutions and supportive policies [215,281]. Appropriate policies at the national and regional level can drive the development of microgrids (as has been the case in the United States [282]). These policies support the integration of microgrids into existing infrastructure and promote the implementation of alternative renewable energy sources [239]. The successful implementation of microgrids also depends on community engagement and acceptance, which can be supported through education and participatory planning [19].

Public–private partnership (PPP) mechanisms play a key role in providing financial and infrastructural support for the implementation of microgrids. Countries such as Germany and India have successfully used PPPs to develop smart microgrid technologies [239].

Ongoing research focuses on increasing the efficiency, scalability and integration of microgrids, with a particular emphasis on overcoming technical, economic and regulatory challenges [19,47]. While the technical, economic and environmental potential of microgrids is well documented, emerging industry needs that focus on cybersecurity, blockchain-based energy markets, dynamic regulation and social justice still need to be addressed. These challenges offer new research opportunities and real-world applications for innovators, developers and policymakers working to accelerate the deployment of microgrids—Table 13. Exploring these areas, which are still being researched, will not only increase the efficiency of microgrids, but also ensure their role in creating sustainable, resilient and inclusive energy systems in the future.

## 8. Conclusions

The evolution of the energy sector towards distributed solutions should not, however, weaken integrated national and continental systems, unless circumstances necessitate it (e.g., distance). These structures can complement and interact within a transformed energy sector, enabling the exchange of energy between areas on market terms. However, this requires a new role for existing distribution and transmission network operators and new functions at the user structure level. An energy manager function may be needed. This is a new business niche for providers of energy solutions—not only technology, but also comprehensive management of the customer’s structure (Energy/Microgrid as a Service). The development of smart energy structures means an increase in demand for sensors and measurement technology—intelligence needs data and its own senses to respond correctly.

Microgrids are not only flexible emergency power supply systems, but they are a new dimension of energy transformation taking place on the consumer side. They are not a concept of disconnecting from the national grid, but a way for the local grid to function as an autonomously managed part, offering flexibility for the entire power system. Microgrids are becoming a key element of the ongoing energy transition, responding to the demand for more sustainable, reliable and decentralised energy systems. By integrating renewable energy sources, microgrids reduce dependence on fossil fuels and greenhouse gas emissions. Advanced technologies, such as smart meters and sensors, control systems and energy management strategies, are an integral part of microgrids, enabling efficient energy distribution and consumption, and helping users achieve energy self-sufficiency.

From the point of view of the user or investor, they have the ability to manage energy production and consumption in order to optimise usage costs, while from the point of view of the energy grid, they can positively influence electricity parameters by providing ancillary services. Microgrids can reduce electricity costs for consumers by optimising the use of renewable energy sources and minimising dependence on the main grid. In addition, they contribute to sustainable environmental development by integrating renewable energy sources and reducing carbon dioxide emissions.

An intelligently managed microgrid, thanks to optimisation algorithms and control strategies, enables independent operation in the event of grid failures or crises, increasing the resilience and reliability of energy systems. Microgrids can co-optimise multiple energy services to ensure system stability. In this case, microgrid generation units, together with energy storage facilities, can ensure frequency stability in the system by providing inertia (including artificial inertia) and participating in regulation services (primary, secondary and tertiary). In addition, in particular, they can increase short-circuit power, ensuring grid rigidity, and, within a local range but affecting the correct operation of the system, regulate reactive power and voltage. This approach is not possible in traditional grids, leading to inefficient energy use and higher operating costs. Furthermore, energy storage systems in microgrids help manage fluctuations in energy generation and ensure a smooth transition between grid-connected and island mode operation. Without these systems, grids cannot effectively vary energy consumption to optimise supply and demand.

A well-designed energy system based on microgrids offers consumers:

- Security of supply even in the event of a national grid failure, and energy self-sufficiency over a longer period of time.
- Stable, predictable energy prices regardless of global crises
- Automatic consumption management through intelligent systems
- The possibility of additional income from energy consumption flexibility.
- In the case of an industrial microgrid—energy security for the company’s continued development, success and innovation.
- Increased property or company assets.
- A clearer conscience thanks to the use of local, green energy.

Microgrids are a transformation technology that uses sensor technologies and promotes energy self-sufficiency, supporting the transition to a more sustainable and resilient energy system. They enable the integration of renewable energy, increase grid reliability, and offer economic and environmental benefits, making them a desirable component of the future energy landscape.

Sensors, being essentially tools for obtaining reliable data, are a critical functional component of microgrids. Their effective use within data collection and interpretation systems, reliability, quality and transfer speed determine the efficiency of microgrid operation. There are several areas in which research and practical applications should be continuously expanded.:

- Integration of advanced AI/ML for sensor data analytics—research can focus on developing AI-based control systems that use sensor data to continuously learn and improve optimisation algorithms, making microgrids more autonomous and adaptive.
- Standardisation of sensor technologies—research is needed to develop standardised sensor platforms that can be universally applied to microgrid systems, facilitating integration and reducing the costs associated with multi-vendor ecosystems.
- Enhancing sensor reliability and durability in harsh environments—research should include more durable, weatherproof sensors that can operate under extreme conditions such as prolonged high temperatures, varying humidity, exposure to dirt and water.
- Cybersecurity for sensor networks—research into secure communication protocols for sensor networks, such as encryption and data authentication, is key to protecting against cyber threats that could compromise the operation of microgrids.
- Optimising energy storage via sensor data—research into the intelligent management of energy storage systems through sensor-based, real-time monitoring could lead to advances in storage efficiency.

Full self-sufficiency of microgrids is only possible with a comprehensive set of components that interact intelligently, autonomously and adapt to local conditions. This includes not only energy generation and storage technologies, but also advanced control, sensor, communication and utility infrastructure.

## Figures and Tables

**Table 1:** Examples of classification criteria for microgrids.

Category	Realisations
Area/application	houses, buildings, campuses, factories, industrial parks, storage halls, agricultural farms, social activity areas, logistics bases, military bases, tourist camps, research and survey stations, vehicles, aircraft and ships
Location	urban, remote
Boundary	static, dynamic
Scenario	residential, industrial, commercial, agriculture, public, special, remote, on-chip, nano-satellite
Energy carriers	electricity: DC, AC (single-phase, three-phase, multi-phase), hybrid (AC/DC); heat and/or cooling, hydrogen, fuels (gaseous, liquid)
Mode of operation	islanded (off-grid), grid-connected
Types of energy sources	emission-free, conventional, mixed
Character	embedded, mobile, temporary
Scale (generation capacity)	small (<10 MW), medium (10–100 MW), large (>100 MW)
Control	centralised, decentralised, distributed
Types of architectures	radial, parallel, star, ring, mesh

Source: own work, based on: [8,15,17,22,38,48,49,50,51,52,53,54].

**Table 2:** Examples of microgrid implementation.

Examples	Fully Autonomous (Off-Grid)	Cooperating with the Power System (On-Grid, Sometimes Off-Grid)
	Objective: to Enable an Independent Power Supply	Objective: Improving Power Quality and Reliability
Covering installation in a single facility	Research stations in hard-to-reach areas, tourist shelters, masts (observation, telecommunications, measurement, etc.), mines, special production plants (close to deposits)	Hospitals, data centres, shopping centres, warehouses, production facilities (e.g., continuous operation), apartment blocks, large-scale buildings, buildings of strategic importance,
Covering multi-site installations	Remote villages, settlements, offshore islands, camps, military bases, outdoor event venues, remote industrial areas, etc.	Campuses/towns (business, university, medical), parks (industrial, warehouse, storage), etc.

Source: own elaboration.

**Table 3:** Modern methods, techniques and instruments for energy management in microgrids.

Methods or Techniques	Instruments
Classical (Linear and Non-linear Programming) Metaheuristics (e.g., Genetic Algorithms, Particle Swarm Optimisation, Grey Wolf Optimisation, Ant Colony Optimisation, Bacterial Foraging Optimisation, Artificial Immune System, Artificial Bee Colony, Crow Search Algorithm) Dynamic programming Stochastic and robust programming Predictive control Multiagent Artificial intelligence (e.g., Fuzzy Logic, Rough sets analysis, Artificial Neural Networks (Deep, Convolutional, Recurrent, etc.), Generative Adversarial Network, Machine Learning, Reinforcement Learning, Deep Learning, Game Theory, Analytic Hierarchy Process, Entropy Weight Method) Other techniques (e.g., based on a Rolling Time Horizon, Q-Iteration Algorithm based on Markov’s Decision Process, Generalised Reduced-Gradient Algorithm, etc.) Blockchain and Internet of Things BIoT ICT	Real-Time Energy Management RTEM Grid-Interactive Efficient Buildings/Object GEBs Demand Side Response DSR ECC (Energy Control Centres): EMS + SCADA Microgrid Control System MCS

Source: own elaboration.

**Table 4:** Types of control within the microgrid management system.

	Centralised	Decentralised	Distributed
Main type of control	Hierarchical	Master-slave	Multi-agent cooperative
Advantages	A globally optimum solution	Communication infrastructure not required High reliability Low computational complexity Higher scalability Acceptable function in real time	Reduced computational complexity Greater reliability Greater scalability Easy function in real time
Disadvantages	Reliable communication infrastructure require Stability dependent on communication network High computational effort Low scalability Complex function in real time	Failure to achieve the global optimum	Suboptimal solution Communication infrastructure required Stability dependent on communication network

Source: own work, based on [8,14,21,53,60,61,62].

**Table 5:** Waste heat recovery technologies.

Devices	Action
Heat exchangers, economisers, waste heat boilers, air heaters	Transferring heat from one medium to another, e.g., from exhaust gases, industrial liquids, air, hot surfaces or waste streams in drying and cooling processes.
Recuperators	Recovery of heat (which would otherwise be lost) from ventilation air (e.g., from production halls, where ventilation exchanges heat with fresh air)
Heat pumps	Use electricity to transport heat from one place to another, capturing heat from the air, water, and ground
Combined heat and power (CHP) systems	Electricity and heat in a single process, significant recovery of waste heat that can be reused in the technological process or for heating
Steam turbines and generators	Recovery of waste heat from industrial processes (process steam) to drive steam turbines with a (synchronous) generator
Absorption cooling systems	Use of waste heat for cooling

Source: own work based on [80,81,82,83].

**Table 6:** Energy storage technologies.

Technology	Features
Short-term energy storage	
Lithium-ion batteries (Li-Ion)	Fast response time (milliseconds), high energy density, decreasing production costs
Other electrochemical cells (BES), e.g., nickel-metal hydride (NiMH), lead-acid (PbA, CLAB), sodium-sulphur (NaS)	Mature technology, relatively short lifetime, high energy density
Supercapacitors (UC, EDLC):	Fast charging and discharging at high power (power fluctuations in the network), very fast response time, long service life (millions of cycles), high instantaneous power
Flywheels (FES)	Store kinetic energy in rotating mass, providing rapid delivery of power pulses (usually for up to several minutes), a source of inertia for the power grid
Thermal energy storage (TES), e.g., molten salts, sand as a heat storage medium	High heat capacity, durability, low storage costs, particularly effective in combination with cogeneration systems, possibility of integration with heating systems
Phase change materials (PCM)	Technologically advanced—the phenomenon of phase change in a material to absorb and release thermal energy
Solid-state batteries	Greater capacity, longer service life than lithium-ion batteries
Other technologies	Flow batteries, including vanadium redox flow batteries (VRFB); superconducting coils (SMES), liquid air LAES
Long-term energy storage	
Hydrogen tanks	Water electrolysis + fuel cells to generate electricity PEM, MCFC, SOFC
Synthetic fuels (ammonia PtA, methane PtN, liquid PtL, methanol)	Storage of large amounts of energy in an easily transportable form, high energy density, retain their energy value for months or years; use in vehicles and as an industrial raw material
CAES compressed air, pressure stores	Storage in tanks/caverns, release as expansion air/gas to drive turbines, possibility of achieving high energy density, energy storage time dependent on the tightness of the installation

Source: own work based on [15,48,84,85,86,87,88,89,90].

**Table 7:** Basic classification of loads in microgrid.

Type	Definition	Examples
Critical load	Loads requiring high quality and reliability of power supply, loads whose failure to operate may cause significant economic, social or health damage.	Loads used in hospitals, military bases, critical infrastructure, security and fire protection installations, water infrastructure or the communications sector
Non-critical load	Load devices that do not require high power supply reliability, as their operation does not affect life-sustaining functions, and their failure to operate at a specific time will not cause significant economic losses or significantly reduce user comfort.	These are usually household goods and appliances such as washing machines, dryers, dishwashers, lighting, air conditioning and heating. In industrial installations, appliances that can be considered non-critical are determined by the specific nature of the production process.

Source: own elaboration.

**Table 8:** Sensors and their use in microgrids.

Sensor Types (Measures)	Examples of Use
Voltage	Monitoring: voltage levels, power quality, network stability and performance, triggering of protective relays, participation in voltage regulation, fault detection, cooperation with Battery Management Systems: Loads [106] Generation [107,108] Energy storage [109,110,111] Energy distribution [112,113,114,115]
Current	Monitoring: power quality, loads, triggering of protections in case of overloads, participation in flow optimisation, fault detection, cooperation with Battery Management Systems: Loads [116,117] Generation [107] Energy storage [23,118] Energy distribution [112,114,115,119,120]
Power (active and reactive)	Smart metering, energy settlement, smart device control, cooperation with EMS, stability assessment, security triggering Loads [121,122,123,124] Generation [124,125,126] Energy storage [124] Energy distribution [115,127,128]
Frequency	In AC systems monitoring of the energy balance, initiation of synchronisation with the external system [129,130,131]
Touch	Manual control of components [132]
Vibration	Equipment diagnostics, identification of maintenance needs Loads [133,134] Generation [135,136,137] Energy storage [138] Energy distribution [139,140]
Acoustic	Equipment diagnostics Loads [141] Generation [142,143,144] Energy distribution [145,146,147]
Temperature	Device diagnostics, support for proper operation, temperature as a control signal Loads [148,149,150,151,152] Generation [153,154,155,156] Energy storage [111,157,158] Energy distribution [159,160]
Speed	Measurement of fluid velocity (water, air, fuels) to improve the efficiency of generation based on the availability of these fluids, Monitoring of infrastructure operating conditions (exposed to wind gusts, for example) [161,162]
Pressure	Internal pressure monitoring in energy storage systems [163,164], in liquid and gas distribution systems, and leak detection
Gas	Monitoring gas emissions, detecting fire hazards, monitoring combustion effects in boiler systems, detecting leaks and other damage and by-products. [165,166]
Humidity	Generation monitoring—humidity affects the performance of PV panels and turbines (including wind turbines) [167,168,169], monitoring in battery rooms—preventing loss of performance or damage
Light	Sunlight intensity as a signal controlling artificial lighting [170], control of generation from solar sources (solar tracking) [171]
Location	Predicting energy consumption according to the location of users and their devices [172,173], estimating the volume of available raw materials (e.g., biomass transport), utilising the available battery potential in EVs, locating equipment and service teams

Source: own elaboration.

**Table 9:** Decarbonisation objectives affecting sustainability that can be pursued by the microgrid.

Objectives	Measures
Cleaner energy	Integration of renewable energy sources and local zero-emission sources EMS—intelligent resource management
Improving energy efficiency	Reduction in network losses EMS—resource optimisation Use of load flexibility
Diversification, energy security	Reduced dependence on centralised infrastructure (national power grid) Own generation and on-site storage Integration of various generation technologies
Reducing energy costs	Own sources—independence from energy markets EMS—price arbitrage
New jobs, services and sources of income	Ancillary services for the power system (resulting from the flexibility of the integrated microgrid structure) Need for an energy manager (in addition to or instead of the company’s chief power engineer) Outsourced energy management and supply—Energy as a Service/Microgrid as a Service (EaaS/MaaS) formula

Source: own elaboration.

**Table 10:** Expected key directions for standardisation of microgrids.

Area of Standardisation	Objective
Definitions, classification, terminology and documentation	Unambiguous identification of network types and their components, consistency of communication between branches
Functionality and interfaces, system performance indicators	Interoperability and reliability of systems, unified criteria for assessing functionality, reliability, power quality, tech-economic evaluation, commissioning, and conformance and acceptance test
Cooperation with sensors	Creating an intelligent, fully automated interoperable structure
Safety requirements	Operational safety and comfort
Scope and equipment requirements	Connection requirements of microgrids to main grid and micro-sources or devices into microgrid; standards and codes addressing the installation of the system; criteria for measuring and expressing the performance of the system, microgrids interconnectivity
Modes of operation	Enabling operations: microgrids black start, coordinated operation with main grid, coordinated operation of multi-microgrids, participation in ancillary service,
Modelling, simulation, design and implementation process	Harmonisation of the technical approach

Source: own elaboration.

**Table 11:** Potential benefits of microgrids.

Character	Examples
Energetic	Improving power supply reliability (security and stability) Controlling energy quality Diversifying power sources Achieving independence Enabling effective energy carrier management (harmonisation with production/core business) Reduction in energy carrier transmission losses Tool for electrification (convenience, efficiency) of economic sectors Opportunity to participate in the energy services market (flexumer)
Financial	Independence from energy carrier markets (own sources) Possibility of additional income from energy flexibility (services for the National Power System—flexumption, generalisation of DSR/DSM): - Active power and frequency regulation (systemic) - Voltage and reactive power regulation (local) - Participation in system restoration (black start) - Power and inertia reserve New forms and models of settlement: - P2P between microgrids—prosumers - Blockchain and dedicated cryptocurrencies (exchangeable for fiat currency) - Outsourcing—EaaS formula (subscription for availability, not for kWh) Lower delivery and generation costs (on-site and from RES)
Environmental	More efficient use of energy: Optimisation of resources Dynamic management of surpluses Reduction in transport of energy carriers Decarbonisation tool: Reduction in pollutant emissions Cleaner air Lower carbon footprint
Organisational and image-related	Environmental responsibility Social responsibility Compliance with ESG criteria Synergy between various industries and the energy sector It is easier to secure (physically and cybernetically) small local microgrids, which are not an attractive target for such attacks anyway Ongoing energy exchange on digital trading platforms
Economy	A place for energy efficiency tools 4D: decarbonisation, diversification, decentralisation, digitalisation Rationalisation of energy carriers and resources New business niches in the energy sector (aggregators, VPPs, EaaS providers, P2P settlement intermediaries—infrastructure/solution providers) A new paradigm for the functioning of the power system: energy from user to user Relief for transmission networks, lower investment requirements

Source: own elaboration.

**Table 12:** Practical challenges in implementing microgrids.

Character	Examples
Technical	Integration with the Main Grid: Synchronisation—microgrids (on-grid mode) must synchronise their frequency and voltage with the main grid to avoid system faults. This requires advanced grid-forming inverters and precise control systems. Bidirectional flow—managing the flow of electricity in both directions between the microgrid and the utility grid requires sophisticated smart grid technologies, capable of real-time load balancing, and fault detection. The need for reliable, efficient, and scalable energy storage systems: Cost and efficiency—current battery storage technologies like lithium-ion batteries are expensive and have limited capacity for long-duration storage. While costs have decreased, they are still a significant component of microgrid investments. Optimal sizing—sizing storage systems is a complex task—too much storage leads to unnecessary cost, while too little compromises system reliability. Dynamic energy management, often using AI-based optimisation algorithms, is essential to balance energy generation, storage, and consumption. State of charge management—advanced energy management systems are required to optimise the charging and discharging cycles of storage systems to maximise efficiency and prolong the lifespan of storage devices. Modularity and scalability are key factors in designing systems that can evolve with changing energy demands: Interoperability of components—integrating various distributed energy resources (DERs) requires high levels of standardisation to ensure compatibility between different manufacturers’ equipment. Communication and control—the Internet of Things (IoT) and edge computing are used for monitoring and control, but ensuring real-time communication between all components, and managing a large number of devices effectively, can be technically challenging. Key role of the management system using data from distributed sensors and intelligent algorithms.
Economic	The initial capital investment and upfront costs of setting up a microgrid system (comprising energy generation, storage, control systems, and infrastructure) can be prohibitively high, especially in areas that lack the necessary financial resources: Infrastructure costs—building or upgrading the local energy infrastructure to support a microgrid requires significant investment in smart meters, controllers, and cabling. Technology costs—advanced technologies for energy storage, control systems, and sensors often come with high acquisition and installation costs. Though prices have been falling for renewable energy systems and batteries, the cost of energy management software and advanced inverters remains relatively high. Although the operating costs of microgrids are generally lower than centralised grids due to reduced transmission losses and fuel consumption, ongoing maintenance and operation costs still pose challenges: Labour costs—regular maintenance of microgrid components (such as solar panels, wind turbines, inverters, etc.) requires skilled labour, which may be scarce in remote or rural areas System upgrades—as new technologies emerge, the microgrid infrastructure may need to be upgraded to incorporate advancements in energy storage, energy management systems, or renewable generation. Such upgrades add to the lifecycle costs. The economic viability of microgrids is closely tied to the payback period on investments. In some cases, the initial investment may take several years to recover, particularly in areas where energy prices are low or government incentives are insufficient. This is a result of high initial costs and the need for substantial energy savings or revenue generation through demand response or energy trading to break even.
Political and regulatory constraints	Regulatory Uncertainty and Policy Support: Permitting and licencing—in many regions, establishing a microgrid requires navigating complex permitting and regulatory processes, including compliance with utility regulations and grid connection standards. The lack of standardised frameworks for microgrid operation in different jurisdictions increases delays and costs. Interconnection rules—the process for connecting a microgrid to the national or regional grid can be time-consuming and costly, particularly when there are differences in grid codes, technical standards, and interconnection requirements. Incentive Structures—while some regions provide government incentives for renewable energy projects, others lack the necessary subsidies or tax incentives to make microgrid investments financially attractive. This lack of consistent policy support can discourage both private and public sector investments in microgrid infrastructure. Energy market structures: Market participation—in many jurisdictions, microgrids are not allowed to participate in energy markets or provide ancillary services like frequency regulation and voltage support. This limits the economic opportunities for microgrids to become financially viable. Tariffs and grid fees—in some cases, microgrid operators may face high fees for using the existing grid infrastructure, or they may be restricted in their ability to sell excess power back to the grid. Political will and local governance: Political instability—in some regions, political instability and lack of long-term policy commitment make it difficult to implement large-scale infrastructure projects like microgrids. Local community buy-in—without local political and community support, microgrid projects can be delayed or cancelled. Strong governance, including transparency and community involvement, is needed to ensure that microgrids meet local energy needs
Social	Public awareness and education: Lack of understanding—many communities are unfamiliar with the technical aspects of microgrids, including how they function, their costs, and their role in ensuring energy security. Cultural acceptance—in some regions, local populations may resist change, particularly if they are accustomed to a centrally managed power grid system. Education campaigns and transparent communication are vital to fostering public support. Stakeholder engagement and social acceptance: Equity and fairness—if not carefully designed, microgrids can exacerbate social inequalities. For example, wealthy communities may disproportionately benefit from the microgrid’s lower energy costs, while poorer, rural communities may not have equal access. Participation in decision-making—ensuring that all relevant stakeholders (including local residents, businesses, and governments) have a voice in the planning and design of microgrid projects is essential for building social acceptance.

Source: own elaboration.

**Table 13:** Issues and directions for desired research.

Description	Need for Research	Industry Needs
Cybersecurity in Multi-Microgrid Ecosystems		
The security implications of interconnected microgrids—especially in regions where multiple microgrids may form a distributed network—remain largely unexplored. In scenarios where multiple microgrids are interconnected to share resources or act as part of a virtual power plant (VPP) or market trading mechanism, the complexity and attack surface of cyber threats increases exponentially	There is a need to develop a multi-layer cybersecurity framework specifically designed for microgrids and their clusters. In this context, attention should be paid to solutions using 5G technology, which allows for the connection of a significantly larger number of sensors and devices per square kilometre, which means more potential entry points for attacks and may also increase the difficulty of managing and monitoring security. Furthermore, the origin of critical 5G components from external suppliers may be a vector for geopolitical or hardware risk.	Real-time threat detection, decentralised security measures and blockchain for secure data transactions and peer-to-peer energy trading are potential areas for enhancing microgrid cybersecurity
Blockchain and Smart Contracts for Distributed Energy Trading		
While blockchain technology has been proposed in the context of energy markets, its practical integration into microgrid systems for peer-to-peer (P2P) energy trading and smart contracts remains underdeveloped. There is a gap in research and industry tools for scalable decentralised energy markets for microgrids.	There is a need to explore how blockchain can enable microgrid users to trade energy directly, bypassing traditional intermediaries. Smart contracts can automate invoicing, billing and dispute resolution, but their legal and technical framework is still at an early stage.	A robust, user-friendly platform for energy transactions, including the integration of microgrids with national or global energy markets, can unlock new revenue streams and opportunities to increase microgrid efficiency
Dynamic Regulation for Autonomous Operations		
Regulatory frameworks often lag behind technological innovation, especially in relation to autonomous microgrid operations and their role in energy markets. Microgrids operating in islanded or hybrid mode face challenges in complying with grid regulations (such as frequency control or voltage regulation) when transitioning between autonomous and grid-connected modes	Research is needed to develop dynamic control models that can facilitate the adaptive principles of real-time microgrids as they autonomously manage generation, storage and load balancing. These models could be based on the principles of flexibility, resilience and transparency in response to real-time system dynamics.	A change in the regulatory framework is needed to allow microgrids to manage themselves, especially in areas prone to frequent power outages or energy crises. The industry requires new guidelines for microgrids that operate outside traditional utility boundaries.
Advanced Fault Detection and Predictive Maintenance for Hybrid Systems		
While fault detection in traditional energy systems is a well-studied area, hybrid microgrid systems—involving multiple sources of generation and storage and complex energy management systems—still face challenges in real-time fault detection, fault prediction and maintenance planning.	More predictive maintenance algorithms, integrated with machine learning, are needed to predict distributed energy resource (DER) failures. The focus should be on detecting early signs of system degradation before they lead to full-scale failures, especially in remote areas where maintenance may require long lead times.	Tools that can provide predictive analysis of system status and automated decision-making for maintenance scheduling would optimise downtime and repair costs, contributing to greater reliability of microgrid operations in different modes of operation
Integrating Microgrids with Demand Side Management (DSM) and Smart Homes		
The integration of microgrids with advanced demand-side management (DSM) and smart home technologies is explored in publications, but usually not in the context of real-time adaptive demand management, in which the microgrid dynamically adjusts energy consumption based on current grid conditions or local generation.	Research is needed into the potential of IoT devices and real-time sensors to enable microgrid-driven DSM, in which smart homes and businesses can dynamically adjust energy consumption based on available local energy or external grid conditions. This can be extended to AI-based learning models that improve energy efficiency and reduce costs over time.	The industry requires software platforms and algorithms capable of connecting home and industrial automation systems directly to the microgrid, enabling automatic scheduling of appliances based on real-time renewable energy availability.
Resilience in Microgrids for Climate Change and Extreme Weather Events		
Climate resilience is a key criterion for larger infrastructure projects, but microgrids designed for extreme weather events—such as hurricanes, floods and fires—still face gaps in design and operational resilience. In particular, there is insufficient research on weather resilience and climate adaptation for renewable-based microgrids.	Research should explore design principles that address specific climate risks in regions prone to extreme events. This could include weatherproof solar panels, wind turbines and battery storage systems that can withstand higher levels of environmental stress.	Industries involved in microgrid development need tools for climate risk assessment and real-time environmental monitoring to ensure that microgrids can adapt to rapidly changing weather patterns and remain operational during disruptions caused by extreme weather events.
Customisation and Standardisation of Microgrid Solutions		
Adapting microgrid solutions to different geographical, cultural and economic contexts is important to achieve widespread adoption, especially in developing countries and remote rural areas. However, the industry lacks standardised approaches to adapting microgrid systems to local needs.	Research is needed to create modular microgrid designs that can be adapted to different climates, energy demands and economic contexts. This includes a study of local resource availability, local grid infrastructure and user preferences for energy services.	The industry needs more open-source platforms that allow users and developers to easily customise microgrid systems, integrate locally available resources and provide affordability with high performance
Energy Equity and Social Impact Evaluation Models		
There is insufficient research on their social impact, particularly in relation to energy equality. Microgrids offer a unique opportunity to empower marginalised communities, but comprehensive models for assessing social impact and ensuring inclusive energy access are still lacking.	New methodologies are needed to assess how microgrids can contribute to social sustainability. This includes the development of impact indicators on job creation, education, health outcomes and community empowerment in microgrid projects.	Energy developers and policymakers need tools to assess the wider societal benefits of microgrids, especially in terms of reducing energy poverty, improving access to healthcare and supporting socio-economic development in underserved areas.

Source: own elaboration.

## Data Availability

No new data were created or analyzed in this study.

## Abbreviations

The following abbreviations are used in this manuscript:

AC	Alternating Current
AI	Artificial Intelligence
CHP	Combined Heat and Power
DC	Direct Current
DER	Distributed Energy Resource
DSM	Demand Side Management
DSR	Demand Side Response
EaaS	Energy as a Service
EMS	Energy Management System
ESG	Environmental, Social, and Governance
ESS	Energy Storage System
EV	Electric Vehicle
GHG	Greenhouse Gas
GPS	Global Positioning System
ICT	Information and Communication Technology
IoT	Internet of Things
P2P	Peer-to-peer
PD	Partial discharge
PMU	Phasor measurements (unit)
PPP	Public–private partnership
PV	Photovoltaic
RES	Renewable Energy Source
SCADA	Supervisory Control and Data Acquisition
VPP	Virtual Power Plant

## References

[1] Cohn L. Microgrid Knowledge. History of Microgrids in the US: From Pearl Street to Plug-and-Play. https://www.microgridknowledge.com/about-microgrids/article/11429549/history-of-microgrids-in-the-us-from-pearl-street-to-plug-and-play.

[2] Wolf G. T&D World. A Short History: The Microgrid. https://www.tdworld.com/digital-innovations/article/20970388/a-short-history-the-microgrid.

[3] Liu Z., Zhang Y., Wang Y., Wei N., Gu C. (2020). Development of the Interconnected Power Grid in Europe and Suggestions for the Energy Internet in China. Glob. Energy Interconnect..

[4] Suryad V.A., Doolla S., Chandorkar M. (2017). Microgrids in India: Possibilities and Challenges. IEEE Electrif. Mag..

[5] Benzaquen J., Miranbeigi M., Kandula P., DIvan D. (2022). Collaborative Autonomous Grid-Connected Inverters: Flexible Grid-Forming Inverter Control for the Future Grid. IEEE Electrif. Mag..

[6] Mullendore S., CleanEnergy Group Energy Resilience Ten Years After Sandy. https://www.cleanegroup.org/energy-resilience-ten-years-after-sandy/.

[7] Du Y., Lu X., Wang X. (2020). Power System Operation with Power Electronic Inverter-Dominated Microgrids. New Technologies for Power System Operation and Analysis.

[8] Uddin M., Mo H., Dong D., Elsawah S., Zhu J. (2023). Microgrids: A Review, Outstanding Issues and Future Trends. Energy Strategy Rev..

[9] World Economic Forum What Are Microgrids—And How Can They Help with Power Cuts?. https://www.weforum.org/stories/2022/05/what-are-microgrids-renewable-power/.

[10] Global Market Insights Microgrid Market Size. https://www.gminsights.com/industry-analysis/microgrid-market.

[11] (2025). Cognitive Market Research Microgrid Market Report. https://www.cognitivemarketresearch.com/microgrid-market-report.

[12] Precedence Research Microgrid Market Size Share Trends 2025 to 2034. https://www.precedenceresearch.com/microgrid-market.

[13] Saeed M.H., Fangzong W., Kalwar B.A., Iqbal S. (2021). A Review on Microgrids’ Challenges & Perspectives. IEEE Access.

[14] Hasan M., Mifta Z., Atia N., Janefar S., Hossain M., Roy P., Chowdhury N., Farrok O. (2023). A Critical Review on Control Mechanisms, Supporting Measures, and Monitoring Systems of Microgrids Considering Large Scale Integration of Renewable Energy Sources. Energy Rep..

[15] Badal F.R., Sarker S.K., Nayem Z., Moyeen S.I., Das S.K. (2023). Microgrid to Smart Grid’s Evolution: Technical Challenges, Current Solutions, and Future Scopes. Energy Sci. Eng..

[16] Syed M.M., Morrison G.M. (2021). A Rapid Review on Community Connected Microgrids. Sustainability.

[17] Nazir M.I., Hussain I., Ahmad A., Khan I., Mallik A. (2022). System Modeling and Reliability Assessment of Microgrids: A Review. Sustainability.

[18] Garcia Vera Y.E., Dufo-Lopez R., Bernal-Agustin J.L. (2019). Energy Management in Microgrids with Renewable Energy Sources: A Literature Review. Appl. Sci..

[19] Almihat M.G.M., Munda J.L. (2025). Comprehensive Review on Challenges of Integration of Renewable Energy Systems into Microgrid. Sol. Energy Sustain. Dev..

[20] Eyimaya S.E., Altin N. (2024). Review of Energy Management Systems in Microgrids. Appl. Sci..

[21] Martin-Martínez F., Sánchez-Miralles A., Rivier M. (2016). A Literature Review of Microgrids: A Functional Layer Based Classification. Renew. Sustain. Energy Rev..

[22] Altaf M.W., Member G.S. (2022). Microgrid Protection Challenges and Mitigation Approaches—A Comprehensive Review. IEEE Access.

[23] Hajiaghasi S., Salemnia A., Hamzeh M. (2019). Hybrid Energy Storage System for Microgrids Applications: A Review. J. Energy Storage.

[24] Kucur G. (2025). Sensors in Microgrids and IoT Technologies. J. Eng. Technol..

[25] Tan K.T., Krishnan S.B., Chua A.Y.Z. (2025). Modelling and Simulation of Pico- and Nano-Grids for Renewable Energy Integration in a Campus Microgrid. Energies.

[26] Marathe M., Venkataramanan G. Picogrid: An Experimental Platform for Prosumer Microgrids. Proceedings of the 2023 IEEE Energy Conversion Congress and Exposition (ECCE 2023).

[27] Kokubu R., Oeda K., Kawakita Y., Yokogawa S., Tobe Y., Ichikawa H. Affinity-Based Power Flow Optimization in Reconfigurable Picogrid. Proceedings of the 2024 IEEE/SICE International Symposium on System Integration, SII 2024.

[28] Ghiani E., Garau M., Celli G., Pilo F., Marongiu G. Smart Integration and Aggregation of Nanogrids: Benefits for Users and DSO. Proceedings of the 2017 IEEE Manchester PowerTech, Powertech 2017.

[29] Burmester D., Rayudu R., Seah W., Akinyele D. (2017). A Review of Nanogrid Topologies and Technologies. Renew. Sustain. Energy Rev..

[30] Sadiku M.N.O., Adebo P.O., Musa S.M., Ajayi-Majebi A. (2021). Nanogrid: An Introduction. Int. J. Eng. Res. Technol..

[31] Lopez Alzate Y., Gómez-Luna E., Vasquez J.C. (2024). Innovative Microgrid Services and Applications in Electric Grids: Enhancing Energy Management and Grid Integration. Energies.

[32] IEC Micro-Grid System. https://syc-se.iec.ch/iec-63097-smart-energy-roadmap/micro-grid-system/.

[33] (2018). IEEE Standard for the Specification of Microgrid Controllers.

[34] Marnay C., Chatzivasileiadis S., Abbey C., Iravani R., Joos G., Lombardi P., Mancarella P., von Appen J. Microgrid Evolution Roadmap. Proceedings of the 2015 International Symposium on Smart Electric Distribution Systems and Technologies (EDST15).

[35] Danley D.R. (2019). Defining a Microgrid Using IEEE 2030.7.

[36] IEEE Power and Energy Society (2017). IEEE Standard for the Specification of Microgrid Controllers.

[37] Ton D.T., Smith M.A. (2012). The U.S. Department of Energy’s Microgrid Initiative. Electr. J..

[38] Hossain A., Roy H., Hossain J., Blaabjerg F. (2019). Evolution of Microgrids with Converter-Interfaced Generations: Challenges and Opportunities. Electr. Power Energy Syst..

[39] Soshinskaya M., Crijns-graus W.H.J., Guerrero J.M., Vasquez J.C. (2014). Microgrids: Experiences, Barriers and Success Factors. Renew. Sustain. Energy Rev..

[40] Dimeas A., Hatziargyriou N. (2005). Operation of a Multiagent System for Microgrid Control. IEEE Trans. Power Syst..

[41] Khan K.R., Siddiqui M.S., Al Saawy Y., Islam N., Rahman A. (2019). Intelligent Monitoring and Control of Microgrid Using Smart Sensor. Procedia Comput. Sci..

[42] Skoczkowski T., Bielecki S., Wołowicz M., Sobczak L., Węglarz A., Gilewski P. (2024). Participation in Demand Side Response. Are Individual Energy Users Interested in This?. Renew. Energy.

[43] Hu J.-L., Bui N.H.B. (2024). The Future Design of Smart Energy Systems with Energy Flexumers: A Constructive Literature Review. Energies.

[44] Schwaegerl C., Tao L., Hatziargyriou N. (2014). The Microgrids Concept. Microgrids. Architectures and Control.

[45] Rodriguez-Gil J.A., Mojica-Nava E., Vargas-Medina D., Arevalo-Castiblanco M.F., Cortes C.A., Rivera S., Cortes-Romero J. (2024). Energy Management System in Networked Microgrids: An Overview.

[46] Ton D. (2009). DOE’s Perspectives on Smart Grid Technology, Challenges, & Research Opportunities. UCLA Engineering SmartGrid Seminar.

[47] Micallef A., Guerrero J.M., Vasquez J.C. (2023). New Horizons for Microgrids: From Rural Electrification to Space Applications. Energies.

[48] Gao D.W. (2015). Energy Storage for Sustainable Microgrid.

[49] Hu J., Islam S., Mareels I., Zhu J., Hu J. (2024). Community Microgrids. Community Energy and Microgrids: Control, Operation and Optimization.

[50] Hatziargyriou N. (2023). Differences and Synergies between Local Energy Communities and Microgrids. Oxford Open Energy.

[51] Muqeet H.A., Javed H., Akhter M.N., Shahzad M., Munir H.M., Nadeem M.U., Sabir S., Bukhari H., Huba M. (2022). Sustainable Solutions for Advanced Energy Management System of Campus Microgrids: Model Opportunities and Future Challenges. Sensors.

[52] Shahbazitabar M., Abdi H., Nourianfar H., Anvari-Moghaddam A., Mohammadi-Ivatloo B., Hatziargyriou N., Anvari-Moghaddam A., Abdi H., Mohammadi-Ivatloo B., Hatziargyriou N. (2021). An Introduction to Microgrids, Concepts, Definition, and Classifications. Microgrids: Advances in Operation, Control, and Protection.

[53] Ferahtia S., Houari A., Cioara T., Bouznit M., Rezk H., Djerioui A. (2024). Recent Advances on Energy Management and Control of Direct Current Microgrid for Smart Cities and Industry: A Survey. Appl. Energy.

[54] Du Y., Men Y., Ding L., Lu X. (2023). Large-Signal Stability Analysis for Inverter-Based Dynamic Microgrids Reconfiguration. IEEE Trans. Smart Grid.

[55] Abdelwanis M.I., Elmezain M. (2024). A Comprehensive Review of Hybrid AC/DC Networks: Insights into System Planning, Energy Management, Control, and Protection. Neural Comput. Appl..

[56] Jung S., Yoon Y.T. (2019). Optimal Operating Schedule for Energy Storage System: Focusing on Efficient Energy Management for Microgrid. Processes.

[57] Duc T.T., Duc T.N., Takano H. (2024). Energy Management of Hybrid AC/DC Microgrid Considering Incentive-Based Demand Response Program. IET Gener. Transm. Distrib..

[58] Tooryan F., Hassanzadehfard H., Collins E.R., Jin S. (2020). Smart Integration of Renewable Energy Resources, Electrical, and Thermal Energy Storage in Microgrid Applications. Energy.

[59] Topa Gavilema O.Á., Álvarez J.D., Torres Moreno J.L., Pérez García M. (2021). Towards Optimal Management in Microgrids: An Overview. Energies.

[60] Mohammed A., Refaat S.S., Bayhan S., Abu-Rub H. (2019). AC Microgrid Control and Management Strategies: Evaluation and Review. IEEE Power Electron. Mag..

[61] Garip S., Bilgen M., Altin N., Ozdemir S. (2023). Reliability Analysis of Microgrids: Evaluation of Centralized and Decentralized Control Approaches. Electr. Power Components Syst..

[62] Amrutha Raju B., Vuddanti S., Salkuti S.R. (2021). Review of Energy Management System Approaches in Microgrids. Energies.

[63] Sun C., Joos G., Ali S.Q., Paquin J.N., Rangel C.M., Al Jajeh F., Novickij I., Bouffard F. (2020). Design and Real-Time Implementation of a Centralized Microgrid Control System with Rule-Based Dispatch and Seamless Transition Function. IEEE Trans. Ind. Appl..

[64] Cárdenas P.A., Martínez M., Molina M.G., Mercado P.E. (2023). Development of Control Techniques for AC Microgrids: A Critical Assessment. Sustainability.

[65] Zhuo W., Savkin A.V., Meng K. (2019). Decentralized Optimal Control of a Microgrid with Solar PV, BESS and Thermostatically Controlled Loads. Energies.

[66] Zuo K., Wu L. (2022). A Review of Decentralized and Distributed Control Approaches for Islanded Microgrids: Novel Designs, Current Trends, and Emerging Challenges. Electr. J..

[67] Peyghami S., Mokhtari H., Blaabjerg F. (2018). Distributed and Decentralized Control of DC Microgrids. DC Distribution Systems and Microgrids.

[68] Wang J., Li K.-J., Javid Z., Sun Y. (2019). Distributed Optimal Coordinated Operation for Distribution System with the Integration of Residential Microgrids. Appl. Sci..

[69] Sarathkumar D., Maheswari P., Manivel M., Jayakumar T., Sivakumar R., Parrthipan B.K. Decentralized and Distributed Control Strategies for Microgrids: A Review of Key Techniques and Applications. Proceedings of the IEEE International Students’ Conference on Electrical, Electronics and Computer Science, SCEECS 2025.

[70] Ding M., Cheng Q., Li L., Bi R., Cao J. (2019). A Design of Central Controller of Microgrid in the Park Based on Embedded System. Power Syst. Prot. Control..

[71] Puradbhat S., Balasubramanian P., Aghamolki H.G. Case Study of the Hierarchical Control Strategies to Maximize the Benefits in Commercial Applications. Proceedings of the IEEE Power Engineering Society Transmission and Distribution Conference.

[72] Zhou B., Zou J., Chung Y.C., Wang H., Liu N., Voropai N., Xu D. (2021). Multi-Microgrid Energy Management Systems: Architecture, Communication, and Scheduling Strategies. J. Mod. Power Syst. Clean Energy.

[73] Mehrjerdi H., Hemmati R., Mahdavi S., Shafie-Khah M., Catalão J.P.S. (2022). Multicarrier Microgrid Operation Model Using Stochastic Mixed Integer Linear Programming. IEEE Trans. Ind. Inform..

[74] Michaelson D., Jiang J. (2021). Review of Integration of Small Modular Reactors in Renewable Energy Microgrids. Renew. Sustain. Energy Rev..

[75] Molina M.G., Mercado P.E. (2018). Renewable Energy Technologies for Microgrids.

[76] França R.P., Monteiro A.C.B., Arthur R., Iano Y. (2021). Overview of Sources of Microgrids for Residential and Rural Electrification: A Panorama in the Modern Age.

[77] Wu J., Chung I.-B., Liu Z., Wang P. (2023). Co-Design Optimization of Combined Heat and Power-Based Microgrids. J. Renew. Sustain. Energy.

[78] Li Y., Huang J., Liu Y., Wang H., Wang Y., Ai X. (2023). A Multicriteria Optimal Operation Framework for a Data Center Microgrid Considering Renewable Energy and Waste Heat Recovery: Use of Balanced Decision Making. IEEE Ind. Appl. Mag..

[79] Ding Z.H., Cao Y.J., Xie L.Y., Lu Y., Wang P. (2019). Integrated Stochastic Energy Management for Data Center Microgrid Considering Waste Heat Recovery. IEEE Trans. Ind. Appl..

[80] Jouhara H. (2021). Waste Heat Recovery in Process Industries.

[81] Hodroj M., Al Takash A., Faraj J., Taher R., Lemenand T., Khaled M. (2024). Comprehensive Review, Categorization, and Practical Insights on Heat Recovery from Hot Air and Exhaust Gas in Engineering Applications. Int. Commun. Heat Mass Transf..

[82] Huang F., Zheng J., Baleynaud J.M., Lu J. (2017). Heat Recovery Potentials and Technologies in Industrial Zones. J. Energy Inst..

[83] Nandhini R., Sivaprakash B., Rajamohan N. (2022). Waste Heat Recovery at Low Temperature from Heat Pumps, Power Cycles and Integrated Systems—Review on System Performance and Environmental Perspectives. Sustain. Energy Technol. Assess..

[84] Boito P., Grena R. (2023). Do We Really Need a Seasonal Energy Storage? Results for Photovoltaic Technology in an Unfavourable Scenario. Renew. Energy Focus.

[85] Qiu Y., Li Q., Ai Y., Wang T., Chen W., Bai H., Benbouzid M., Liu S., Gao F. (2024). Optimal Scheduling for Microgrids Considering Long-Term and Short-Term Energy Storage. J. Energy S.

[86] Kusko A., Dedad J. (2007). Stored Energy—Short-Term and Long-Term Energy Storage Methods. IEEE Ind. Appl. Mag..

[87] Faias S., Santos P., Sousa J., Castro R. (2008). An Overview on Short and Long-Term Response Energy Storage Devices for Power Systems Applications. Renew. Energy Power Qual. J..

[88] Ruchika R., Jain D.K. (2023). A Brief Review on Hybrid Energy Storage Systems for Microgrid Application. Prz. Elektrotechniczny.

[89] Kurkus-Gruszecka M., Krawczyk P., Badyda K. (2024). Operation Algorithms of Seasonal Thermal Storage in the Conditions of a District Heating Company. Rynek Energii.

[90] Zwierzchowski R., Wołowicz M. (2020). Energy and Exergy Analysis of Sensible Thermal Energy Storage—Hot Water Tank for a Large CHP Plant in Poland. Energies.

[91] Driesen J., Katiraei F. (2008). Design for Distributed Energy Resources. IEEE Power Energy Mag..

[92] Watson J.D., Ojo Y., Laib K., Lestas I. (2021). A Scalable Control Design for Grid-Forming Inverters in Microgrids. IEEE Trans. Smart Grid.

[93] Jafari H., Poursalan A., Gholami A., Gavagsaz-Ghoachani R., Phattanasak M. A Review of Solar Tracking Technologies: Mechanisms, Challenges, and Future Directions. Proceedings of the 2024 International Conference on Materials and Energy: Energy in Electrical Engineering (ICOME-EE).

[94] Duong H.-N., Tran L., Vu T., Vo-Duy T., Nguyễn B.-H. (2024). A Global Optimal Benchmark for Energy Management of Microgrid (GoBuG) Integrating Hybrid Energy Storage System. IEEE Trans. Smart Grid.

[95] Zhang M., Xu Q., Zhang C., Nordström L., Blaabjerg F. (2022). Decentralized Coordination and Stabilization of Hybrid Energy Storage Systems in DC Microgrids. IEEE Trans. Smart Grid.

[96] Sharma P., Sharma A. A Review on Flywheel Energy Storage System in Microgrid. Proceedings of the 2022 2nd International Conference on Advance Computing and Innovative Technologies in Engineering (ICACITE).

[97] Deng Y., Luo F., Zhang Y., Mu Y. (2023). An Efficient Energy Management Framework for Residential Communities Based on Demand Pattern Clustering. Appl. Energy.

[98] Khan K.R., Rahman A., Nadeem A., Siddiqui M.S., Khan R.A. Remote Monitoring and Control of Microgrid Using Smart Sensor Network and Internet of Thing. Proceedings of the 1st International Conference on Computer Applications and Information Security, ICCAIS 2018.

[99] Bertocco M., Giorgi G., Narduzzi C., Tramarin F. A Case for IEEE Std. 1451 in Smart Microgrid Environments. Proceedings of the SMFG 2011—IEEE International Conference on Smart Measurements for Grids.

[100] Liyon Raj S., Malleeshwaran T., Prasanna T., Alfred Daniel J. Smart Energy Optimization Harnessing IoT and Advanced Learning Techniques. Proceedings of the 4th International Conference on Sustainable Expert Systems, ICSES 2024—Proceedings.

[101] Agarwal Y., Weng T., Gupta R.K. (2011). Understanding the Role of Buildings in a Smart Microgrid. Proceedings—Design, Automation and Test in Europe.

[102] Asghar E., Hill M., Lynch C. A Review of Data-Driven Smart Energy Management Systems for Distribution Networks. Proceedings of the 2023 IEEE PES GTD International Conference and Exposition, GTD 2023.

[103] Perka B., Suproniuk M., Piwowarski K. (2019). Application of Acoustic Surface Wave to Measure Busbar Temperature [Zastosowanie Akustycznej Fali Powierzchniowej Do Pomiaru Temperatury Szyn Prądowych]. Prz. Elektrotechniczny.

[104] Najafirad M.J., Dehkordi N.M. (2023). Distributed Event-Triggered Control of DC Microgrids with Output Saturation Constraint. Int. J. Electr. Power Energy Syst..

[105] Ye X., Wang Z., Wang S. (2025). Data-Driven Voltage Control in Isolated AC Microgrids Subject to Sensor Saturation. Appl. Sci..

[106] Katalin Á. (2019). Voltage Monitoring and Supply Controlling System. Procedia Manuf..

[107] Swapna V., Gayatri M.T.L. Power Quality Issues of Grid Integration of Distributed Generation: A Review. Proceedings of the 2021 International Conference on Computational Performance Evaluation, ComPE 2021.

[108] Thiruburasundari V., Ramya G., Ramadevi N., Pameela M. (2023). Power Quality Concerns Due to Power System Improvements with Renewable Energy Sources and Its Remedies. J. Trends Comput. Sci. Smart Technol..

[109] Parqui W.O.R., Danielsson G.H., Da Paixao J.L., Sperandio M. Real-Time Co-Simulation of Voltage Control in Battery Energy Storage Systems for Distribution Networks with High Photovoltaic Penetration. Proceedings of the 2024 Workshop on Communication Networks and Power Systems, WCNPS 2024.

[110] Xue Z., Dong B., Zhang Y. (2022). Monitoring and Management Technical Research for Battery Energy Storage. Lect. Notes Electr. Eng..

[111] Xue Z., Dong B. Design and Implementation of Monitoring and Management System for Battery Energy Storage. Proceedings of the 2022 IEEE 5th International Electrical and Energy Conference, CIEEC 2022.

[112] Milanović J., Bollen M., Čukalevski N. Guidelines for Monitoring Power Quality in Contemporary and Future Power Networks—Results from CIGRE/CIRED JWG C4.112. Proceedings of the CIGRE Session 45—45th International Conference on Large High Voltage Electric Systems 2014.

[113] Geyer R., Diendorfer C., Knöttner S., Drexler-Schmid G., Hasan A.S.M.M.S.M.M., Tuhin R.A., Ullah M., Sakib T.H., Thollander P., Trianni A. (2020). Eceee Industrial Summer Study Proceedings. Eceee Ind. Summer Study Proc..

[114] Sapountzoglou N., Raison B., Silva N. Fault Detection and Localization in LV Smart Grids. Proceedings of the 2019 IEEE Milan PowerTech, PowerTech 2019.

[115] Esreraig M., Mitra J. An Observer-Based Protection System for Microgrids. Proceedings of the IEEE Power and Energy Society General Meeting.

[116] Volodarsky E., Voloschko A. (2014). Monitoring System of Electricity Quality in Decentralized Electricity Supply Systems. Eastern-Eur. J. Enterp. Technol..

[117] Popa G.N. (2022). Electric Power Quality through Analysis and Experiment. Energies.

[118] Wang Y., Tian J., Sun Z., Wang L., Xu R., Li M., Chen Z. (2020). A Comprehensive Review of Battery Modeling and State Estimation Approaches for Advanced Battery Management Systems. Renew. Sustain. Energy Rev..

[119] Herrada A., Orozco-Henao C., Pulgarín Rivera J.D., Mora-Flórez J., Marín-Quintero J. (2025). Fault Detection System for Smart City Distribution Networks: A Long Short-Term Memory-Based Approach. Energies.

[120] Su H., Mu C., Wang N., Liu J. (2013). Method of Micro-Grid Protection. J. Cent. South. Univ. Sci. Technol..

[121] Mari S., Bucci G., Ciancetta F., Fiorucci E., Fioravanti A. (2022). A Review of Non-Intrusive Load Monitoring Applications in Industrial and Residential Contexts. Energies.

[122] Akinci T.C., Sengezer E., Dursun E., Yilmaz M., Gokmen G., Martinez-Morales A.A., Penchev M., Raju A.S.K. Smart Meter Analytics for Residential Energy Efficiency. Proceedings of the IEEE Global Energy Conference 2024, GEC 2024.

[123] Palacios-García E.J., Guan Y., Savaghebi M., Vásquez J.C., Guerrero J.M., Moreno-Munoz A., Ipsen B.S. Smart Metering System for Microgrids. Proceedings of the IECON 2015—41st Annual Conference of the IEEE Industrial Electronics Society.

[124] Uribe-Pérez N., Hernández L., de la Vega D., Angulo I. (2016). State of the Art and Trends Review of Smart Metering in Electricity Grids. Appl. Sci..

[125] Brandao D.I., Simões M.G., Farret F.A., Antunes H.M.A., Silva S.M. (2018). Distributed Generation Systems: An Approach in Instrumentation and Monitoring. Electr. Power Compon. Syst..

[126] Rocabert J., Luna A., Blaabjerg F., Rodríguez P. (2012). Control of Power Converters in AC Microgrids. IEEE Trans. Power Electron..

[127] Abdel-Majeed A., Tenbohlen S., Schöllhorn D., Braun M. (2013). Meter Placement for Low Voltage System State Estimation with Distributed Generation. IET Conference Publications.

[128] Subba R.B., Umanand L. Development of Energy Monitoring System for a Typical Micro-Grid. Proceedings of the e-Energy’19: The Tenth ACM International Conference on Future Energy Systems.

[129] Blaabjerg F., Teodorescu R., Liserre M., Timbus A. (2006). V Overview of Control and Grid Synchronization for Distributed Power Generation Systems. IEEE Trans. Ind. Electron..

[130] Booma J., Meenakshi Sundaram B., Suresh S., Karthikeyan K. (2025). A Novel Decentralized Dynamic State Estimation Methodology for Effective Frequency Monitoring in Smart Grids. J. Chin. Inst. Eng. Trans. Chin. Inst. Eng. A.

[131] Seok K.-H., Ko J., Park C.-W., Kim C.-H., Kim Y.S. (2013). Distributed Sensor Network-Based Virtual FDR System. Int. J. Distrib. Sens. Netw..

[132] Titus J. (2011). Thumbs up for Touch Sensors: Touch Sensors Can Replace Mechanical Switches, but Fi Rst You Must Understand Noise, Materials, and Software. ECN Electron. Compon. News.

[133] Egaji O.A., Ekwevugbe T., Griffiths M. A Data Mining Based Approach for Electric Motor Anomaly Detection Applied on Vibration Data. Proceedings of the World Conference on Smart Trends in Systems, Security and Sustainability, WS4 2020.

[134] Ribeiro Junior R.F., Areias I.A.S., Gomes G.F. (2021). Fault Detection and Diagnosis Using Vibration Signal Analysis in Frequency Domain for Electric Motors Considering Different Real Fault Types. Sens. Rev..

[135] Wang Y., Sun F.-X., Huang T.-S. Development of On-Line Vibration Condition Monitoring System of Hydro Generators. Proceedings of the 2004 International Conference on Machine Learning and Cybernetics.

[136] Narasinh V., Mital P., Chakravortty N., Mittal S., Kumar A.V., Venkatraman C., Kulkarni N., Thakur I. (2024). Analysing and Forecasting Degradation in Wind Turbines under Transient Operating Conditions through Vibration Analysis. Proceedings of the E3S Web of Conferences.

[137] Barkova N., Barkov A., Grishchenko D. (2019). Vibration Diagnostics of Equipment Units with Gas Turbine Engines. Vibroengineering Procedia.

[138] Hasegawa T., Saeki M., Ogawa T., Nakano T. (2019). Vibration-Based Fault Detection for Flywheel Condition Monitoring. Procedia Struct. Integr..

[139] Nezhad A., Samimi M. (2025). A Review of Vibration-Based Techniques for the Condition Assessment and Failure Detection of Transformers. J. Vib. Eng. Technol..

[140] Sikora A., Walłecki M., Karp Ł. (2008). Diagnosis of the Operational Status of Electromechanical Devices with an Optical Fiber Vibration Sensor. Metrol. Meas. Syst..

[141] Orman M., Pinto C.T. Usage of Acoustic Camera for Condition Monitoring of Electric Motors. Proceedings of the IEEE Region 10 Annual International Conference, Proceedings/TENCON.

[142] Kerkyras S., Karakassidis V., Papaelias M. Condition Monitoring of Wind Turbine Gearboxes Using Acoustic Emission. Proceedings of the 10th International Conference on Condition Monitoring and Machinery Failure Prevention Technologies 2013, CM 2013 and MFPT 2013.

[143] Ramírez I.S., Márquez F.P.G., Sánchez P.J.B., Papaelias M., Malik H. (2025). Maintenance Management Based on Aerial Acoustic Monitoring for Offshore Wind Turbines. Lect. Notes Data Eng. Commun. Technol..

[144] Sanchez P.J.B., Ramirez I.S., Marquez F.P.G. Wind Turbines Acoustic Inspections Performed with UAV and Sound Frequency Domain Analysis. Proceedings of the 2021 7th International Conference on Control, Instrumentation and Automation, ICCIA 2021.

[145] Cefer V., Kucera M., Gutten M., Brncal P., Jarina R. Visualisation and Diagnostics of Acoustic Emission from Transformers. Proceedings of the 2020 International Conference on Diagnostics in Electrical Engineering, Diagnostika 2020.

[146] Gialluca G.T., Gialluca G.T., Masiero B., De Lima E.R., Almeida L.M., Fruett F. A Wireless Weatherproof Acoustic Sensor System to Detect Anomalies in Substation Power Transformers. Proceedings of the 36th SBC/SBMicro/IEEE/ACM Symposium on Integrated Circuits and Systems Design, SBCCI 2023.

[147] Filho O.G.S., Brasil F.S., Neto F.F.S., Leite R.C., Zaghetto S.L. (2012). Application of Acoustic Emission to High-Voltage Electric Power Equipment Diagnostics. Nondestruct. Test. Mater. Struct..

[148] Wang J., Tang Y., Schenato L. (2024). Humans-in-the-Building: Getting Rid of Thermostats in Comfort-Based Energy Management Control Systems. IEEE Control Syst. Lett..

[149] Matsui K. HVAC Control System as a Home Energy Management System Function to Prevent Heat Shock in Households. Proceedings of the 2016 IEEE International Conference on Smart Grid Communications, SmartGridComm 2016.

[150] Šebök M., Gutten M., Kučera M., Korenčiak D., Kołtunowicz T.N. (2014). Nondestructive Diagnostics of Electrical Systems and Equipments. Prz. Elektrotechniczny.

[151] Hey J., Malloy A.C., Martinez-Botas R., Lamperth M. (2016). Online Monitoring of Electromagnetic Losses in an Electric Motor Indirectly through Temperature Measurement. IEEE Trans. Energy Convers..

[152] Sebok M., Gutten M., Kucera M., Korenciak D. (2020). Condition Analysis of Electrical Machines by Thermovision. Prz. Elektrotechniczny.

[153] Buratto W.G., Muniz R.N., Nied A., Barros C.F., Cardoso R., Gonzalez G.V. (2024). A Review of Automation and Sensors: Parameter Control of Thermal Treatments for Electrical Power Generation. Sensors.

[154] González I., Folgado F.J., Calderón A.J. (2022). Visualisation and Analysis of Digital and Analog Temperature Sensors in PV Generator through IoT Software. Eng. Proc..

[155] Jankovec M., Topič M. (2013). Intercomparison of Temperature Sensors for Outdoor Monitoring of Photovoltaic Modules. J. Sol. Energy Eng. Trans. ASME.

[156] Kandry H., Ennawaoui C., Laadissi E.M., Loualid E.M., El Ballouti A., Malki Z., El Jouad M., Balhamri A., Hajjaji A. (2022). Optimized Photovoltaic Panels Power Using Cooling System Based Thermoelectric Materials. Mater. Today Proc..

[157] Liu H. Research on the Application of Intelligent Sensors in Temperature Monitoring and Prediction in New Energy Storage Power Systems. Proceedings of the 2024 5th International Symposium on New Energy and Electrical Technology, ISNEET 2024.

[158] Zheng Y., Che Y., Hu X., Sui X., Stroe D.-I., Teodorescu R. (2024). Thermal State Monitoring of Lithium-Ion Batteries: Progress, Challenges, and Opportunities. Prog. Energy Combust. Sci..

[159] Guo Y., Du B.X., Gao Y., Li X., Li H.B. On-Line Monitoring System Based on MODBUS for Temperature Measurement in Smart Grid. Proceedings of the 2012 IEEE Innovative Smart Grid Technologies—Asia, ISGT Asia 2012.

[160] Lvov M.Y., Nikitina S.D., Lesiv A. (2023). V Using Thermal Indicators to Monitor the Condition of Electrical Contacts and Connections during the Operation of Electrical Equipment. Power Technol. Eng..

[161] Braquehais J.E.D.P., Lisboa De Souza A.A. Energy-Autonomous Wind Speed Smart Sensor. Proceedings of the Conference Record—IEEE Instrumentation and Measurement Technology Conference.

[162] Mishra A., Kumar R., Khalkho A.M., Mohanta D.K. An IoT Integrated Reliability Estimation of Wind Energy System. Proceedings of the 2022 International Conference on IoT and Blockchain Technology, ICIBT 2022.

[163] Huang Z., Wang H., Yang T., Li C., Li H. (2022). Simulation of Internal Temperature and Pressure for Cells in an Energy Storage Lithium-Ion Battery. Power Syst. Prot. Control.

[164] Lai X., Yu J., Mao S., Han X., Zhu Z., Zheng Y., Wang Y., Ouyang M. (2025). Enhancing Early Warning Systems: Experimental Investigation of Physical Signals in Thermal Runaway Evolution of Large-Capacity Lithium Iron Phosphate Batteries. J. Power Sources.

[165] Karthikeyan S., Pandya H.M., Sharma M.U., Gopal K. (2015). Gas Sensors—A Review. J. Environ. Nanotechnol..

[166] Nazemi H., Joseph A., Park J., Emadi A. (2019). Advanced Micro- and Nano-Gas Sensor Technology: A Review. Sensors.

[167] Danook S.H., Jassim K.J., Hussein A.M. (2019). The Impact of Humidity on Performance of Wind Turbine. Case Stud. Therm. Eng..

[168] Ayadi F., Colak I., Genc N., Bulbul H.I. Impacts of Wind Speed and Humidity on the Performance of Photovoltaic Module. Proceedings of the 8th International Conference on Renewable Energy Research and Applications, ICRERA 2019.

[169] Kazem H.A., Chaichan M.T. (2015). Effect of Humidity on Photovoltaic Performance Based on Experimental Study. Int. J. Appl. Eng. Res..

[170] Sowa S. (2018). Improving the Energy Efficiency of Lighting Systems by the Use of Solar Radiation. Proceedings of the E3S Web of Conferences.

[171] Paliyal P.S., Mondal S., Layek S., Kuchhal P., Pandey J.K. (2024). Automatic Solar Tracking System: A Review Pertaining to Advancements and Challenges in the Current Scenario. Clean Energy.

[172] Shaptala R., Kyselova A. Location Prediction Approach for Context-Aware Energy Management System. Proceedings of the 2016 IEEE 36th International Conference on Electronics and Nanotechnology, ELNANO 2016.

[173] Lee W., Chang-Sung J. (2022). Low Power Sensor Location Prediction Using Spatial Dimension Transformation and Pattern Recognition. Energies.

[174] Diefenderfer P., Jansson P.M., Prescott E.R. Application of Power Sensors in the Control and Monitoring of a Residential Microgrid. Proceedings of the SAS 2015—2015 IEEE Sensors Applications Symposium.

[175] Hosseinzadeh N., Mousavi A., Teirab A., Varzandeh S., Al-Hinai A. Real-Time Monitoring and Control of a Microgrid—Pilot Project: Hardware and Software. Proceedings of the 2019 29th Australasian Universities Power Engineering Conference, AUPEC 2019.

[176] Vargas-Salgado C., Aguila-Leon J., Chiñas-Palacios C., Hurtado-Perez E. (2019). Low-Cost Web-Based Supervisory Control and Data Acquisition System for a Microgrid Testbed: A Case Study in Design and Implementation for Academic and Research Applications. Heliyon.

[177] Prasad P.S.D.S., Pavankumar Y., Manikanta G.S.S.N.S., Reddy O.J. Fault Detection Method for Inverter Interfaced Distributed Generators Based Microgrid. Proceedings of the 2024 2nd International Conference on Smart Technologies for Power and Renewable Energy, SPECon 2024.

[178] Mao J., Yin C., Zhang X., Wu A., Zhang X. (2022). Learning Observer-Based Sensor Fault-Tolerant Control of Distributed Generation in an Islanded Microgrid for Bus Voltage Stability Enhancement. Sensors.

[179] Hussain G.A., Zaher A.A., Hummes D., Safdar M., Lehtonen M. (2020). Hybrid Sensing of Internal and Surface Partial Discharges in Air-Insulated Medium Voltage Switchgear. Energies.

[180] Badicu L.-V., Koltunowicz W., Broniecki U., Batlle B. Increased Operation Reliability through PD Monitoring of Stator Winding. Proceedings of the 13th International Electrical Insulation Conference, INSUCON 2017.

[181] Baker P.C., Judd M.D., McArthur S.D.J. (2010). A Frequency-Based RF Partial Discharge Detector for Low-Power Wireless Sensing. IEEE Trans. Dielectr. Electr. Insul..

[182] Gao K., Lyu L., Huang H., Fu C., Chen F., Jin L. Insulation Defect Detection of Electrical Equipment Based on Infrared and Ultraviolet Photoelectric Sensing Technology. Proceedings of the IECON 2019—45th Annual Conference of the IEEE Industrial Electronics Society.

[183] Ma L., Li Z., Yang S., Wang J. (2025). A Review on Vibration Sensor: Key Parameters, Fundamental Principles, and Recent Progress on Industrial Monitoring Applications. Vibration.

[184] Bogue R. (2013). Sensors for Condition Monitoring: A Review of Technologies and Applications. Sens. Rev..

[185] Srikanth T., Chitra Selvi S. (2022). A Novel Centralized Supervisory with Distributed Control System-Based Microgrid. Automatika.

[186] Xiao Z. (2023). DC Microgrids for Electric Vehicle Wireless Charging.

[187] Chauhan K., Chauhan R.K. (2017). Optimization of Grid Energy Using Demand and Source Side Management for DC Microgrid. J. Renew. Sustain. Energy.

[188] Jurenoks A., Jokić D. (2017). Sensor Network Information Flow Control Method with Static Coordinator within Internet of Things in Smart House Environment. Procedia Comput. Sci..

[189] Wang X., Liang Q. (2018). Efficient Sensor Selection Schemes for Wireless Sensor Networks in Microgrid. IEEE Syst. J..

[190] Hilleringmann U., Petrov D., Mwammenywa I., Kagarura G.M. Local Power Control Using Wireless Sensor System for Microgrids in Africa. Proceedings of the IEEE AFRICON.

[191] Cullen J., Cherchi C., Perciavalle P., Johnson K., Jacangelo J.G. (2019). Operations, Asset Management and Data Analytics Nexus—A Closed Loop Function Approach. Proceedings of the WEFTEC 2019—92nd Annual Water Environment Federation’s Technical Exhibition and Conference.

[192] Su X., Cai Z., Jia X., Guo L., Ding Z. (2020). A Self-Adaptive Approach for Mobile Wireless Sensors to Achieve Energy Efficient Information Transmission. IEEE Access.

[193] Lin Y., Vokkarane V.M., Zahidul Islam M., Edib S.N. (2024). Resilient Sensing and Communication Architecture for Microgrid Management.

[194] Sharmila N., Nataraj K.R., Rekha K. An Overview on Design and Control Schemes of Microgrid. Proceedings of the 2019 Global Conference for Advancement in Technology, GCAT 2019.

[195] Lu X., Wang J. (2017). A Game Changer: Electrifying Remote Communities by Using Isolated Microgrids. IEEE Electrif. Mag..

[196] Beshley P., Kaidan M., Strykhalyuk B., Kochan O., Mokhun S., Fedchyshyn O. IoT Empowered SmartESS Systems for Real-Time Monitoring and Control in Smart Grid. Proceedings of the 5th IEEE International Conference on Advanced Information and Communication Technologies, AICT 2023.

[197] Senthilkumar G., Mallala B., Sivarajan S., Harish C., Harsha D., Natrayan L. Maximizing Power Utilization through Machine Learning and IoT Based Power Flow Strategies in DC Micro Grids with Renewable Energy Resources. Proceedings of the 7th International Conference on Inventive Computation Technologies, ICICT 2024.

[198] Krit S., Benaddy M., El Habil B., Laassiri J., Said E.H. Reliability of Transport Data and Energy Efficient in Wireless Sensor Networks: A Literature Survey. Proceedings of the 2016 International Conference on Engineering and MIS, ICEMIS 2016.

[199] Ghosh P.K., Sahinoglu M., Phoha V. V Resource Management for Uninterrupted Microgrid Operation. Proceedings of the 2019 the 7th International Conference on Smart Energy Grid Engineering, SEGE 2019.

[200] Gao J., Wu R., Hao J., Xu C. (2023). Energy Scheduling for Solar-Powered Isolated Microgrids with Intelligent Monitoring Based on WSN. Electr. Power Constr..

[201] Hoummadi M.A., Aroussi H.A., Bossoufi B., Karim M., Mobayen S., Zhilenkov A., A H., Alghamdi T. (2024). A Review of Constraints and Adjustable Parameters in Microgrids for Cost and Carbon Dioxide Emission Reduction. Heliyon.

[202] Fan Y., Zhang L., Li D., Wang Z. (2023). Progress in Self-Powered, Multi-Parameter, Micro Sensor Technologies for Power Metaverse and Smart Grids. Nano Energy.

[203] Kantsepolsky B., Aviv I. Quantum Sensing for the Cities of the Future. Proceedings of the International Archives of the Photogrammetry, Remote Sensing and Spatial Information Sciences—ISPRS Archives.

[204] Maharjan S., Jayatissa A.H. (2024). A Review of Flexible Sensors.

[205] Lee S., Lee H., Jung G., Kwak M.S., Kim Y.-R., Ko H. (2025). Artificial Flexible Sensory Electronics Mimicking Human Somatosensory System. Korean J. Chem. Eng..

[206] Ahn J., Cho S., Wu L., Li X., Lee D., Ha J.-H., Han H., Lee K., Kang B., Kwon Y. (2024). Innovations in Self-Powered Sensors Utilizing Light, Thermal, and Mechanical Renewable Energy. Nano Energy.

[207] Zheng Y., Cao L.-X., Lv J.-R., Wen H.-Y., Mao L.-X., Wang X.-Q., He Z.-Z. (2025). Self-Powered Flexible Sensor Network for Continuous Monitoring of Crop Micro-Environment and Growth States. Meas. J. Int. Meas. Confed..

[208] Peter N., Gupta P., Goel N. (2025). Intelligent Strategies for Microgrid Protection: A Comprehensive Review. Appl. Energy.

[209] Li H., Johra H., de Andrade Pereira F., Hong T., Le Dréau J., Maturo A., Wei M., Liu Y., Saberi-Derakhtenjani A., Nagy Z. (2023). Data-Driven Key Performance Indicators and Datasets for Building Energy Flexibility: A Review and Perspectives. Appl. Energy.

[210] Cheng G., Lin Y., Abur A., Gomez-Exposito A., Wu W. (2024). A Survey of Power System State Estimation Using Multiple Data Sources: PMUs, SCADA, AMI, and Beyond. IEEE Trans. Smart Grid.

[211] Yehia D.M., Numair M., Mansour D.-E.A. (2024). Novel IoT-Based Droop Control for Battery SoC Balancing Among Multiple Microgrids. IEEE Trans. Smart Grid.

[212] Ye Z.-J., Izadi M., Farajollahi M., Mohsenian-Rad H. (2024). A Remedy to Losing Time Synchronization at D-PMUs, H-PMUs, and WMUs in Event Location Identification in Power Distribution Systems. IEEE Trans. Smart Grid.

[213] Bangali S., Athanasios V., Dimitrios L., Arya P., Singhai J., Das S. (2024). Smart Meter-Based Outage Detection and Fault Location in a Microgrid in Gaidouromantra, Greece. IET Conf. Proc..

[214] Dahlan N.Y., Ahmad N., Ilham N.I., Yusoff S.H. (2022). Energy Security: Role of Renewable and Low-Carbon Technologies.

[215] Shahzad S., Abbasi M.A., Ali H., Iqbal M., Munir R., Kilic H. (2023). Possibilities, Challenges, and Future Opportunities of Microgrids: A Review. Sustainability.

[216] Caceres-Najarro L.A., Jung J., Lee Y., Yoo S., Salman M., Kim J., Lee G., Noh Y. (2024). Towards Energy Independence at KENTECH: A Comprehensive Microgrid Implementation Roadmap. Heliyon.

[217] Khazaei J., Schlauderaff C. (2019). Data on Reducing Carbon Footprint in Microgrids Using Distributed Battery Energy Storage. Data Br..

[218] Danish F., Shamsi M.F., Kumar A., Siddiqui A.S., Sarwar M. Impact Assesment of Microgrid Towards Achieving Carbon Neutrality: A Case Study. Proceedings of the 2023 International Conference on Power, Instrumentation, Energy and Control (PIECON).

[219] Kaur A., Kumar P., Kaur J. (2025). Small Scale Renewable Energies and Storage for Microgrids.

[220] Li M., Zhang X., Li G., Jiang C. (2020). A Feasibility Study of Microgrids for Reducing Energy Use and GHG Emissions in an Industrial Application. Appl. Energy.

[221] Mehigan L., Al Kez D., Collins S., Foley A., Ó’Gallachóir B., Deane P. (2020). Renewables in the European Power System and the Impact on System Rotational Inertia. Energy.

[222] Torto S.O.G., Pachauri R.K., Singh J.G., Khan B., Ali A. (2024). Networked Micro-Grid Topologies for Transactive Energy Management System: An Overview and Future Perspectives. Sci. Afr..

[223] Zou H., Mao S., Wang Y., Zhang F., Chen X., Cheng L. (2019). A Survey of Energy Management in Interconnected Multi-Microgrids. IEEE Access.

[224] Bielecki S., Skoczkowski T., Sobczak L., Wołowicz M. (2022). Electricity Usage Settlement System Based on a Cryptocurrency Instrument. Energies.

[225] Prasad H., Bhadane K.V., Kulkarni H.R. Recent Control and Integration Issue of Distributed Energy Resources in Smart Microgrid: A Review. Proceedings of the 1st International Conference on Innovative Trends and Advances in Engineering and Technology, ICITAET 2019.

[226] Abubakar A.M., Itamah E.I., Batool K., Avsar C., Asif M., Kutarov V.V., Coto B., Abbas K. (2025). Advancing Sustainable Energy: Integrating Small-Scale Renewables and Storage in Microgrids. Energy Management Systems for Microgrids with Wind, PV and Battery Storage.

[227] Fu Q., Hamidi A., Nasiri A., Bhavaraju V., Krstic S.B., Theisen P. (2013). The Role of Energy Storage in a Microgrid Concept: Examining the Opportunities and Promise of Microgrids. IEEE Electrif. Mag..

[228] Abdelsattar M., Ismeil M.A., Aly M.M., Abu-Elwfa S.S. (2024). Analysis of Renewable Energy Sources and Electrical Vehicles Integration Into Microgrid. IEEE Access.

[229] Naseri N., El Hani S., El Harouri K., Mediouni H. (2022). Primary and Secondary Control of an Autonomous Solar Microgrid Based Power-to-X: Renewable Hydrogen Conversion. Int. J. Hydrogen Energy.

[230] Vivas F.J., Segura F., Andújar J.M., Palacio A., Saenz J.L., Isorna F., López E. (2020). Multi-Objective Fuzzy Logic-Based Energy Management System for Microgrids with Battery and Hydrogen Energy Storage System. Electronics.

[231] Li Z., Zheng Z., Xu L., Lu X. (2019). A Review of the Applications of Fuel Cells in Microgrids: Opportunities and Challenges. BMC Energy.

[232] Kumar A., Jain P., Sharma S. (2022). Transactive Energy Management for Microgrids Considering Techno-Economic Perspectives of Utility–a Review. Int. J. Energy Res..

[233] De Sena A.S., Ullah M., Nardelli P.H.J. (2023). Edge Computing in Smart Grids. Handbook of Smart Energy Systems.

[234] Alaguraj R., Kathirvel C. Edge Computing and IoT-Driven Renewable Energy Integration for Decentralized Smart Grid Optimization. Proceedings of the International Conference on Circuit Power and Computing Technologies, ICCPCT 2024.

[235] Hong G., Hanjing C. An Edge Computing Architecture and Application Oriented to Distributed Microgrid. Proceedings of the 19th IEEE International Symposium on Parallel and Distributed Processing with Applications, 11th IEEE International Conference on Big Data and Cloud Computing, 14th IEEE International Conference on Social Computing and Networking and 11th IEEE International Conference on Sustainable Computing and Communications, ISPA/BDCloud/SocialCom/SustainCom 2021.

[236] Sarathkumar D., Bashkaran K., Manivel M., Jayakumar T., Sivakumar R., Anandhakumar C. An Extensive Critique on Advances in Digital Control Techniques for Smart Grid and Micro Grid Operation. Proceedings of the 2025 IEEE International Students’ Conference on Electrical, Electronics and Computer Science, SCEECS 2025.

[237] Chenaru O., Toma L., Mîndra T., Simin Ş., Fleancu Ş. (2021). Development of Smart Microgrids, a Smart Grid Base; [Dezvoltarea Mictroreţelelor Inteligente, Baza Reţelelor Inteligente]. Energy Environ. Effic. Resour. Glob..

[238] Kim M. (2013). Toward Smart Microgrid with Renewable Energy: An Overview of Network Design, Security, and Standards. International Conference on Computational Science and Its Applications.

[239] Almihat M.G.M., Munda J.L. (2025). The Role of Smart Grid Technologies in Urban and Sustainable Energy Planning. Energies.

[240] Zulu M.L.T., Sarma R., Tiako R. (2025). Enhancing Power Quality in a PV/Wind Smart Grid with Artificial Intelligence Using Inverter Control and Artificial Neural Network Techniques. Electricity.

[241] Rezaee Jordehi A., Mansouri S.A., Tostado-Véliz M., Hakimi S.M., Safaraliev M., Nasir M. (2025). A Three-Level Model for Integration of Smart Homes, Electric Vehicle Charging Stations and Hydrogen Fuelling Stations in Reconfigurable Microgrids Considering Vehicle-to-Infrastructure (V2I) Technology. Energy.

[242] Sun Y., Meng Y., Ge L., Zhang Y., Wang S., Wang J. (2023). Application and Prospect of Microgrid Operation Optimization Enabled by Artificial Intelligence. High Volt. Eng..

[243] Monchusi B.B., Mokwana T.I. A Review of Advancements in AI-Based Control Techniques for Microgrids. Proceedings of the International Conference on Electrical, Computer, Communications and Mechatronics Engineering, ICECCME 2024.

[244] Pandin M., Syahlani N., Utomo A.W., Sumaedi S., Hidayat M., Gunawan H., Yarmen M., Prihanto I.G. Trends and Insights in Artificial Intelligence Applications for Microgrid Management: A Bibliometric Analysis. Proceedings of the 2024 International Conference on Computer, Control, Informatics and its Applications (IC3INA).

[245] Patel T.A., Subbarao S., Vikas S., Vimal K.A., Paul A., Smaran A., Sinchana G., Yadunandan K., Shubhachethan, Akarsh Application of AIML and IOT for Reliable Microgrid Installed at Billenahoshalli and Lakshmanapura by Nie-Crest. Proceedings of the 8th IEEE International Conference on Computational System and Information Technology for Sustainable Solutions, CSITSS 2024.

[246] Bilal M., Algethami A.A., Imdadullah, Hameed S. (2024). Review of Computational Intelligence Approaches for Microgrid Energy Management. IEEE Access.

[247] Hadi M., Elbouchikhi E., Zhou Z., Saim A., Shafie-khah M., Siano P., Rahbarimagham H., Colom P.M. (2025). Artificial Intelligence for Microgrids Design, Control, and Maintenance: A Comprehensive Review and Prospects. Energy Convers. Manag. X.

[248] Umar A., Kumar D., Ghose T. (2022). Blockchain-Based Decentralized Energy Intra-Trading with Battery Storage Flexibility in a Community Microgrid System Point of Common Coupling Network Administrator or Network Provider Peak to Peak Average Ratio Supply to Demand Ratio. Appl. Energy.

[249] Ali L., Azim M.I., Peters J., Bhandari V., Menon A., Tiwari V., Green J., Muyeen S.M. (2023). Application of a Community Battery-Integrated Microgrid in a Blockchain-Based Local Energy Market Accommodating P2P Trading. IEEE Access.

[250] Mahesh G.S., Babu G.D., Rakesh V.G.T., Mohan S.B., Ranjit P.S. Energy Management with Blockchain Technology in DC Microgrids. Proceedings of the International Conference on Materials and System Engineering (ICMSE).

[251] Vivek M.U., Selvaprabhu P. (2022). Role of telecommunication technologies in microgrids and smart grids. Smart Grids and Microgrids: Technology Evolution.

[252] Zhang L., Cao Y., Zhang G., Huang Y., Chen S. (2022). Blockchain-Based Secure Data Sharing Scheme for Microgrid. Comput. Eng..

[253] Syamsuddin S., Manjang S., Nappu M.B., Paundu A.W. (2025). AI-Enhanced Hybrid PoW/PoS Consensus for Secure and Energy-Efficient Blockchain Microgrids. Eng. Technol. Appl. Sci. Res..

[254] Evens M., Ercoli P., Arteconi A. (2024). On the Application of Blockchain Technology in Microgrids. Commun. Comput. Inf. Sci..

[255] Wang J., Wang Q., Zhou N., Chi Y. (2017). A Novel Electricity Transaction Mode of Microgrids Based on Blockchain and Continuous Double Auction. Energies.

[256] Zhuo D., Chunlong L., Zhipeng Y., Yang C., Yuan J. (2018). Demand Analysis of Wireless Sensor Networks for New Energy Micro-Grid. IOP Conf. Ser. Mater. Sci. Eng..

[257] Griego D., Schopfer S., Henze G., Fleisch E., Tiefenbeck V. (2019). Aggregation Effects for Microgrid Communities at Varying Sizes and Prosumer-Consumer Ratios. Energy Procedia.

[258] Ghiani E., Pilo F., Soma G.G., De Tuglie E.E., Cagnano A., Conti S. (2020). Case studies of microgrids systems. Microgrids for Rural Areas.

[259] Rädle S., Mast J., Gerlach J., Bringmann O. Exploration of Computational Intelligence Strategies for Increasing the Degree of Self-Sufficiency of Microgrids. Proceedings of the NEIS 2020—Conference on Sustainable Energy Supply and Energy Storage Systems.

[260] Bidram A., Reno M.J., Abadi S.A.G.K., Aparicio M.J., Bauer D. (2024). Trends in Dc Microgrids: From the Control and Protection Perspective. IEEE Electrif. Mag..

[261] Abdolahzadeh A., Hassannia A., Mousavizadeh F., Tolou Askari M. (2025). Multi-Objective Stochastic Model Optimal Operation of Smart Microgrids Coalition with Penetration Renewable Energy Resources with Demand Responses. Sci. Rep..

[262] Kim J., Oh H., Choi J.K. (2022). Learning Based Cost Optimal Energy Management Model for Campus Microgrid Systems. Appl. Energy.

[263] Van Der Mei A.J., Doomernik J.-P. (2020). Perfect Storm for Monopoly Grids II: The Dual Disruptive Impact of Distributed Generation and Local Competition. CIRED.

[264] Chauhan R.K., Chauhan K., Singh S.N. (2020). Microgrids for Rural Areas: Research and Case Studies.

[265] Einabadi N., Kazerani M. (2025). Nanogrids in Modern Power Systems: A Comprehensive Review. Smart Cities.

[266] Zhang X., Sharma R., He Y. Optimal Energy Management of a Rural Microgrid System Using Multi-Objective Optimization. Proceedings of the 2012 IEEE PES Innovative Smart Grid Technologies, ISGT 2012.

[267] Singh S.K., Sharma A., Mishra S., Srivastava S.C., Mukherjee D., Srivastava A., Schulz N. IEEE A Rural Microgrid Field Pilot in India Ensuring Reliable Electricity Supply and Social Upliftment. Proceedings of the 2021 IEEE Global Humanitarian Technology Conference (GHTC).

[268] Suri D., Shekhar J., Mukherjee A., Singh Bajaj A. Designing Microgrids for Rural Communities: A Practitioner Focused Mini-Review. Proceedings of the 2020 IEEE International Conference on Environment and Electrical Engineering and 2020 IEEE Industrial and Commercial Power Systems Europe, EEEIC/I and CPS Europe 2020.

[269] Bhatta R., Shrestha R., Negri C., Schmitt K., Musraf M., Chamana M., Illham O., Bayne S. Feasibility of a Real-World Test Microgrid Facility to Provide Economic and Resiliency Benefits in Extreme Weather Conditions. Proceedings of the 2022 IEEE Power and Energy Society Innovative Smart Grid Technologies Conference, ISGT 2022.

[270] De M., Das G., Mandal K.K. (2021). Proposing Intelligent Energy Management Model for Implementing Price Rate in Microgrids Using Demand Response Program. J. Inst. Eng. Ser. B.

[271] Patel R., Paleari W., Selva D. Architecture Study of an Energy Microgrid. Proceedings of the 2016 11th Systems of Systems Engineering Conference, SoSE 2016.

[272] An J., Hong T. (2025). Optimizing Battery Energy Storage System Placement in Energy Intensive Cities for Sustainable Urban Development Considering Multiple Objectives. Sustain. Cities Soc..

[273] Nesihath M.K., Aneesha M.V., Nafeesa K., Sreedharan S. Optimization and Energy Management of Microgrid Considering Demand Response. Proceedings of the International Conference on Futuristic Technologies in Control Systems and Renewable Energy, ICFCR 2024.

[274] Neeraj, Gupta P., Tomar A. Optimization of Energy Storage Systems for Efficient Energy Management in Microgrid. Proceedings of the 2023 IEEE 2nd International Conference on Industrial Electronics: Developments and Applications, ICIDeA 2023.

[275] Avilés A.C., Oliva H.S., Watts D. (2019). Single-Dwelling and Community Renewable Microgrids: Optimal Sizing and Energy Management for New Business Models. Appl. Energy.

[276] Arıkan Yildiz Y., Güçyetmez M., Aktemur C. (2025). 3E Analysis of a Wind-Solar-Biomass Energy Generation in Context of Regional Multi-Microgrids: A Case Study of Sivas, Türkiye. Renew. Energy.

[277] Rabocha T., Maslii O., Robochyi V., Frolov O., Pizintsali L. (2023). Ukraine’s Energy Supply in the Defense Sector: The First Lessons of War. Sustain. Eng. Innov..

[278] Kurbatova T., Sidortsov R., Trypolska G., Hulak D., Sotnyk I. (2024). Maintaining Ukraine’s Grid Reliability under Rapid Growth of Renewable Electricity Share: Challenges in the Pre-War, War-Time, and Post-War Periods. Int. J. Sustain. Energy Plan. Manag..

[279] Keen S., Ayres R.U., Standish R. (2019). A Note on the Role of Energy in Production. Ecol. Econ..

[280] Skoczkowski T., Bielecki S., Wołowicz M. (2025). Redefining Energy Management for Carbon-Neutral Supply Chains in Energy-Intensive Industries: An EU Perspective. Energies.

[281] Chartier S.L., Venkiteswaran V.K., Rangarajan S.S., Collins E.R., Senjyu T. (2022). Microgrid Emergence, Integration, and Influence on the Future Energy Generation Equilibrium—A Review. Electronics.

[282] Feng W., Jin M., Liu X., Bao Y., Marnay C., Yao C., Yu J. (2018). A Review of Microgrid Development in the United States—A Decade of Progress on Policies, Demonstrations, Controls, and Software Tools. Appl. Energy.

